# Isotope dilution LC-MS/MS for the quantification of ergot alkaloids: a comparative study

**DOI:** 10.1007/s00216-026-06581-4

**Published:** 2026-06-02

**Authors:** Sven-Oliver Herter, Susanne Krentscher, Kevin Sassin, Christian Kornrumpf, Sarah Kulas, Hajo Haase, Matthias Koch

**Affiliations:** 1 Division 1.7 Organic Trace and Food Analysis, Bundesanstalt für Materialforschung und -prüfung (BAM), Richard-Willstätter-Str. 11, 12489 Berlin, Germany; 2 Eurofins WEJ Contaminants GmbH, Neuländer Kamp 1, 21079 Hamburg, Germany; 3 Department of Food Chemistry and Toxicology, Technische Universität Berlin, Kaiserin-Augusta-Allee 14, 10553 Berlin, Germany

**Keywords:** HPLC-MS/MS, Ergot alkaloids, Internal standard, Cereals, Reference material

## Abstract

Ergot alkaloids (EAs) are toxic secondary metabolites formed by various fungi, most notably *Claviceps purpurea*, which infects cereal crops such as rye, wheat, and barley. Due to their toxicity, the European Commission established maximum levels for 12 priority EAs in cereals and related products in 2022. Routine monitoring, typically employing high-performance liquid chromatography coupled to tandem mass spectrometry, is challenged by the unavailability of isotope-labeled internal standards. To address this, we previously synthesized internal standards (ISTDs) for 12 priority EAs and herein we evaluate their performance through an interlaboratory comparison against the standard addition approach prescribed in EN 17425. The ISTDs significantly improved method precision and trueness, with results in good agreement across both laboratories. Furthermore, we developed a reference material for EAs in rye flour, in accordance with ISO 33405 guidelines, using stable isotope dilution mass spectrometry as primary method. This internal RM contributes to safer foodstuffs by supporting reliable method development and validation for EAs.

**Graphical abstract:**

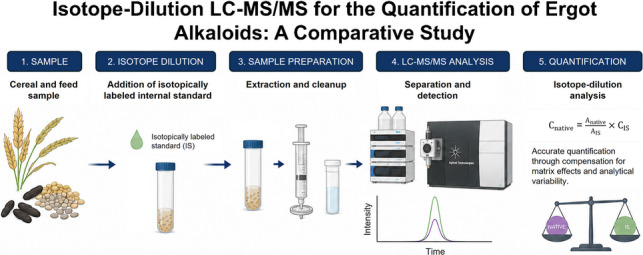

**Supplementary Information:**

The online version contains supplementary material available at 10.1007/s00216-026-06581-4.

## Introduction

Ergot alkaloids (EAs) are a diverse group of naturally occurring toxins produced by different fungi. The most common species in Europe is *Claviceps purpurea*, which mainly infects cereal crops such as rye, wheat, and barley, though it can also affect rice, maize, sorghum, oats, and millet [1, 2]. During infection, the fungus invades the host plant and replaces the developing grain with a dark, hardened fungal mycelium called sclerotium. Historically, EAs were known for their toxic effects and were responsible for widespread poisoning events in medieval Europe, collectively known as “St. Anthony’s Fire” or ergotism, and are among the earliest recorded mass foodborne poisonings in human history [3]. Symptoms of ergotism include hallucinations, convulsions, and severe cases of gangrene caused by restricted blood flow [4, 5]. Today, mechanical separation techniques are commonly used to remove most of the sclerotia from harvested grain. However, smaller fragments and abrasions are often not removed and lead to the introduction of EAs into the food chain [6, 7].

**Fig. 1:**
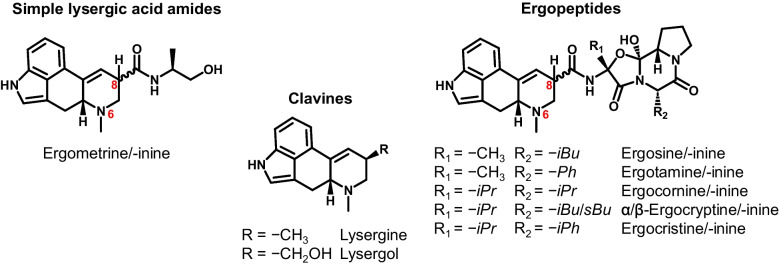
Representative structures of the three major groups of EAs: simple lysergic acid amides, clavines, and ergopeptines (R: *-iPr* (isopropyl), *-iBu* (isobutyl), -*sBu* (sec-butyl), *-Ph* (phenyl)). The locants highlight the *C*^*8*^-atom, where the epimerization occurs, and the *N*^*6*^-atom, where the isotope-labeled methyl group is attached to in the ISTD

Chemically, EAs are tryptophan-derived indole alkaloids and can be classified into three major structural classes based on the substituents attached to the ergoline scaffold: clavines, simple lysergic acid amides, and ergopeptines (Fig. 1). The core structure of most EAs is based on ergoline, a tetracyclic skeleton. Clavines, which include structures such as lysergine and lysergol, represent the simplest class of EAs. Both simple lysergic acid amides and ergopeptines are derived from D-lysergic acid and differ primarily in the complexity of the substituent attached to the amide bond. In simple amides, this substituent is typically an alkyl amide, whereas in ergopeptines, the structure is more complex, featuring a cyclic tripeptide moiety based upon L-proline [8, 9]. Ergopeptines and simple lysergic acid amides are susceptible to epimerization at the *C*^*8*^-atom when exposed to factors such as intense light, elevated pH, heat, or protic solvents. This leads to a mixture of the *8R-*epimer which is called the “-*ine*” form and the *8S*-epimer which is called the “-*inine*” form. Both forms can interconvert into each other and vary in biological activity, although the *8S*-epimer has shown to be generally less biologically active [10, 11].

To evaluate the potential health risk for the civilian population, the European Food Safety Authority (EFSA) was assigned by the European Commission to deliver a scientific opinion on EAs in food and feed. Based on the reported data and calculations, the EFSA recommended a tolerable daily intake of 0.6 µg/kg body weight per day for the sum of the 12 priority EAs in 2012 [12]. They represent the most common EAs produced by *Claviceps purpurea* and include the epimeric pairs: ergometrine/ergometrinine, ergotamine/ergotaminine, ergosine/ergosinine, ergocornine/ergocorninine, ergocryptine/ergocryptinine (α- and β-form), and ergocristine/ergocristinine (c.f. Fig. 1). Despite this, analytical surveys have reported levels in some rye-based products exceeding 7000 µg/kg and suggesting that dietary exposure, especially among children, can surpass the tolerable daily intake under certain conditions [12, 13].

In response to an increased awareness of EAs in food- and feed samples, the European Commission implemented maximum levels for the sum of the 12 priority EAs as of January 1, 2022 [14]. These values apply to unprocessed cereal grains, processed milling products, wheat gluten, and infant cereals, ranging from 500 µg/kg for rye-based products down to 20 µg/kg for processed cereal-based food for infants and young children. Furthermore, the EU proposed a reduction by half of the maximum limits for rye and wheat products, intended to take effect on July 1 st, 2024 [15]. However, following consultations with member states and relevant stakeholders, the EU determined that the proposed lower limits are not yet achievable, and their implementation has been postponed until July 1 st, 2028 [16].

Alongside the introduction of maximum levels in 2022, the European Committee for Standardization (CEN) Technical Committee 275 developed the European Norm (EN) 17425:2021 [17]. It describes a method for the determination of EAs in cereals and cereal products using high-performance liquid chromatography (HPLC) coupled to tandem mass spectrometry (MS/MS). HPLC-MS/MS has become increasingly common in routine laboratories during the last decades and has largely replaced older methods such as HPLC with fluorescence detection for the quantification of EAs [18]. However, two main challenges in HPLC-MS/MS analysis are differences in extraction efficiency between samples and matrix effects that influence measurement accuracy [19, 20]. To correct for matrix effects, EN 17425 applies a single-point standard addition (SA) procedure, which calculates recovery rates (RR) and corrects the quantification results accordingly.

However, when available, an isotope-labeled internal standard (ISTD) provides a more robust alternative [21–23]. ISTDs have the same chemical structure as the target analyte but are enriched with stable isotopes such as ^2^H, ^13^C, ^15^N and ^18^O. Isotopologues containing ^13^C, ^15^N, or ^18^O exhibit the same retention time as the target analyte. In contrast, ^2^H-labeled compounds elute earlier in reversed-phase chromatography due to the increased polarity of the carbon deuterium bond. Although retention times are similar, the analyte and the ISTD can be clearly distinguished in mass spectrometric analysis because of their different mass-to-charge ratio (*m/z*). This principle is used in stable isotope dilution analysis mass spectrometry (SIDA-MS), in which the ISTD is added in equal amounts to each calibration solution and sample prior to extraction and analysis [24, 25]. In SIDA, both the ISTD and the analyte undergo the same variations during sample extraction, cleanup, measurement, and the quantification is achieved by calculating the ratio of the analyte signal to the ISTD signal. The analyte-to-ISTD peak area ratio is evaluated using a calibration curve, constructed from serial dilutions of a standard into known concentrations of the native compound, spiked with the ISTD. Although absolute signal intensities may vary due to extraction efficiency between aliquots or during measurement, the analyte-to-ISTD peak area ratio remains stable. Importantly, the Consultative Committee for Amount of Substance (CCQM) recognizes SIDA as a primary ratio method of measurement. In mass spectrometry, when certified native reference standards are used for calibration, the results and uncertainties obtained with SIDA-MS are fully traceable to the International System of Units (SI), providing the highest levels of accuracy and metrological reliability in mass spectrometry–based analysis [26].

At the time we started our work, isotope-labeled standards for the quantification of the priority EAs were not scientifically or commercially available. To address this limitation, we developed a simple semisynthetic procedure to synthesize the required ^13^CD_3_-ISTD from the unlabeled EA, as described in our recent publications [27, 28].

The present study had two objectives. First, we evaluated the performance of the EN 17425 SA procedure in comparison with the ISTD-based approach via a cross-laboratory study in collaboration with Eurofins WEJ Contaminants GmbH (Hamburg, Germany). After demonstrating the performance of the ISTDs on different contaminated samples, we screened rye flour purchased from local supermarkets to produce a reference material (RM). The characterization of the RM was carried out following ISO 33405:2024 [29]. The study assessed the homogeneity and stability of the RM and estimated the combined uncertainty. This RM provides an essential tool for performance monitoring and supports the validation of analytical methods for EA detection and quantification in rye flour and related products.

## Materials and methods

### Chemicals and equipment

All chemicals were used without further purification. The native dried down standards for all priority EAs (ergometrine/-inine, ergosine/-inine, ergotamine/-inine, ergocornine/-inine, α-ergocryptine/-inine, and ergocristine/-inine) were purchased from RomerLabs Division Holding GmbH (Tulln, Austria). Acetonitrile (LCMS-grade) was purchased from Th. Geyer (Renningen, Germany) and ultrapure water was produced with a Purelab flex deionization system (Veolia Holding Deutschland GmbH, Hamburg, Germany). Ammonium carbonate (99.999%, metal basis) and Ammonium hydroxide solution (≥ 25%) for LC-MS were purchased from Fischer Scientific GmbH (Hampton, NH, USA). Bondesil-PSA was purchased from Agilent Technologies Inc. (Waldbronn, Germany). 1 mL Luer-Slip syringes were purchased from B. Braun SE (Melsungen, Germany) and the syringe filters Chromafil Xtra PTFE (13 mm, 0.2 µm) were obtained from Macherey-Nagel (Düren, Germany).

For the addition of the extraction solvent a 50 ml automatic tilting pipet (Glaswarenfabrik Karl Hecht GmbH & Co. KG, Sondheim vor der Rhön, Germany) was used. The samples were shaken on a horizontal shaker (Ika Werke GmbH & Co. KG., Staufen im Breisgau, Germany) and the samples were centrifuged with a 6k15 laboratory centrifuge from Sigma Laborzentrifugen GmbH (Osterode am Harz, Germany).

Quantification was performed on an Infinity II HPLC system equipped with a G7167B autosampler, a G7120A binary pump, a G7116B thermostat coupled to a G6465B Ultivo Triple Quadrupole mass spectrometer (Agilent, Waldbronn, Germany). The separation was performed on a Phenomenex Kinetex EVO C_18_-column (150 mm × 2.1 mm, 1.7 µm).

### Flour and bread samples

Eurofins provided two different rye flour, two wheat flour, and two bread samples (shredded into 0.1–0.5 mm sized pieces) for the laboratory cross comparison study which were stored at −20°C until analysis. For the screening of rye flour, a total of 10 samples were purchased from different local German supermarkets.

### Reference material preparation

The aim was to produce a rye flour RM with a total mass fraction for the 12 priority EAs between 250 µg/kg and 300 µg/kg. To achieve this, we first surveyed rye flours from local German supermarkets. Each sample was tested individually, and those with suitable EA content were combined (total 7.7 kg) and thoroughly mixed for 72 h in a drum hoop mixer.

After homogenization, six 125 mL wide-neck amber glass bottles with screw caps were each filled with 50 g of flour taken from different positions in the drum to assess the homogeneity. Additionally, eleven 125 mL wide-neck amber glass bottles with screw caps were filled with 50 g of flour for the isochronous stability study. The remaining RM was stored in a barrel at − 20 °C.

### Calibrators

The dried down reference standards were reconstituted gravimetrically in accordance with the manufacturer’s specifications. Subsequent dilutions of the stock solutions were carried out volumetrically in a suitable volumetric flask to obtain a native working solution with a concentration of 500 ng/mL for each of the native priority EAs. The dried down isotope-labeled mix standard contained 2500 ng/mL of ^13^CD_3_-ergometrine/-inine, ^13^CD_3_-ergosine/-inine, ^13^CD_3_-ergotamine/-inine, ^13^CD_3_-ergocornine/-inine, ^13^CD_3_-α-ergocryptine/-inine, and ^13^CD_3_-ergocristine/-inine and was volumetrically reconstituted with acetonitrile:water (90 V%:10 V%) and diluted in a 5 mL volumetric flask with acetonitrile to obtain a 500 ng/mL isotope-labeled working solution.

Calibrators were prepared from the native and isotope-labeled working solution in a volumetric flask at different concentration levels of 30, 10, 5, 2, 1, 0.2, 0.1 ng/mL (corresponds to 150, 50, 25, 10, 5, 1, 0.5 µg/kg, respectively) for the native EAs and with a constant concentration level of 2 ng/mL (10 µg/kg) for the isotope-labeled EAs.

### Sample extraction

10.000 g ± 0.050 g of a flour or bread sample was weighted into a 75 mL screw-cap glass centrifuge tube. For the preparation of the spiked samples used in the SA or ISTD approach, 200 µL of the 500 µg/mL native working solution or isotope-labeled working solution was added to 10 g sample and allowed to dry at room temperature for 1 h. The extraction solvent was prepared in accordance with EN 17425:2021, consisting of acetonitrile:water (84 v%:16 v%) with 200 mg/L ammonium bicarbonate, adjusted to pH 9 using concentrated ammonium hydroxide solution. 50 mL of the extraction solvent was added to each centrifuge tube using an automatic tilting pipette. The samples were extracted by shaking horizontally at 300 rpm for 30 min, followed by centrifugation at 3500 rpm (2931 *g*, 4 °C) for 10 min. 1 mL of the supernatant was transferred into a 4 mL amber glass vial containing 50 mg ± 5 mg of the Bondesil-PSA sorbent. The vial was capped tightly and shaken horizontally at 300 rpm for 2 min. The extract was drawn into a plastic Luer slip syringe and filtered through a 13 mm PTFE (0.22 µm) syringe filter into a 2 mL amber glass vial for HPLC-MS/MS analysis.

### HPLC-MS/MS parameters

Four microliters of the extract was injected into the system and separation was achieved on a Phenomenex Kinetex EVO C_18_ column at 40 °C in gradient mode at a flow rate of 0.3 mL/min. Mobile phase A consisted of ultrapure water with 200 mg/L ammonium bicarbonate adjusted to pH 9 using concentrated ammonium hydroxide solution and phase B consisted of acetonitrile without additives. The gradient program is provided in Table 1.

**Table 1:** Gradient program for the separation of EAs on a Phenomenex Kinetex EVO C_18_ (150 mm × 2.1 mm, 1.7 µm) column

Time [min]	H_2_O + 200 mg/L (NH_4_)_2_CO_3_	Acetonitrile
0.00	95%	5%
0.50	95%	5%
3.00	55%	45%
10.00	25%	75%
10.10	0%	100%
13.00	0%	100%
13.10	95%	5%
16.50	95%	5%

The HPLC system was coupled to a triple quadrupole mass spectrometer from Agilent with an electrospray ionization (ESI) source. The ESI source was operated in positive ionization mode with the following parameters: capillary voltage 2500 V, gas temperature 250 °C, gas flow 10 L/min, nebulizer 25 psi, sheath gas temperature 375 °C, sheath gas flow 12 L/min and nozzle voltage 0 V. The individual MS/MS parameters and retention times are given in supplementary information (SI) Table S1 for each of the unlabeled and isotope-labeled EAs.

### Recovery rates

The RR for the SA method ${RR}_{SA}$ at BAM was determined using Eq. 1 with ${w}_{s}$ representing the measured mass fraction of the spiked sample in µg/kg; ${w}_{u}$ the mass fraction of the unspiked sample in µg/kg; and ${w}_{L}$ the mass fraction added to the spiked sample, here 10 μg/kg.

1 $$\begin{array}{c}{RR}_{SA} [\%]=\frac{{w}_{s}-{w}_{u}}{{w}_{L}}\times 100\end{array}$$

For the ISTD method at BAM, the RR was calculated as the ratio of the ISTD peak area from the sample to the average ISTD peak area of the calibration standards. Eurofins reported the RRs determined for each individual analyte using both the SA and ISTD methods. These data were then used to calculate the mean RR and the standard deviation.

### Limit of detection and limit of quantification

The limit of detection (LOD) and limit of quantification (LOQ) were determined using the calibration curve method based on DIN 32645:2008. Therefore, ten equidistant calibration points in a concentration range of 200–2 ng/L (corresponds to 1000–1 ng/kg, respectively) were measured in triplicates and the *LOD* and *LOQ* were calculated based on Eqs. 2 and 3:

2 $$\begin{array}{c}LOD=s_{x;0}\times t_{f;\alpha}\times\sqrt{\frac1m+\frac1n+\frac{\overline x}{Q_x}}\end{array}$$

3 $$\begin{array}{c}LOQ=k\times s_{x;0}\times t_{f;\frac\alpha2}\times\sqrt{\frac1m+\frac1n+\frac{\left[\left(3\times LOD\right)-\overline x\right]^2}{Q_x}}\end{array}$$

with $s_{x;0}\approx\frac{S_{y,x}}b$ the standard deviation of the procedure (*s*_*y,x*_ = standard deviation of the residuals of the calibration samples and *b* = slope of the calibration curve); *t*_*f;α*_ = quantile of the t-distribution for a one-sided test and ${t}_{f;\frac{\alpha }{2}}$ = quantile of the t-distribution for a two-sided test with f = n − 2 degrees of freedom; *n* = number of calibration samples; *m* = number of measurements on the calibration sample; $\overline x$ = arithmetic mean of the mass concentration of the calibration samples; ${Q}_{x}$ = sum of squared differences of the individual calibration levels from the mean calibration level; *1/k* = relative uncertainty of the result used to characterize the detection limit (33.3% at *k* = 3).

### Drum homogeneity study

The drum homogeneity study evaluates the distribution of the priority EAs in the homogenized RM, allowing the calculation of the preliminary uncertainty contribution from potential heterogeneity (between-unit inhomogeneity) to be included in the RM overall uncertainty budget. For the homogeneity test, 50 g flour was taken from six different positions within the drum and analyzed for all priority EAs. Each sample was extracted in triplicate following the extraction and analytical methods described above and all 18 extracts were analyzed in a randomized order under repeatability conditions and quantified using the same calibration.

The analyte-specific inhomogeneity contribution to the overall uncertainty budget of the RM was estimated in accordance with ISO 33405 using two models. The between-unit standard deviation ${s}_{bu}$ was determined using Eq. 4, while the minimum inhomogeneity contribution between units ${s}_{bu,min}$ was calculated using Eq. 5, as specified in Annex C of ISO 33405. The term ${s}_{bu,min}$ approximates the maximum level of variability that may remain undetected due to insufficient repeatability or method variability.

4 $$\begin{array}{c}{s}_{bu}=\sqrt{\frac{{M}_{between}-{M}_{within}}{n}}\end{array}$$

5 $$\begin{array}{c}{s}_{bu,min}=\sqrt{\frac{{M}_{within}}{n}}\times \sqrt[4]{\frac{2}{N\times \left(n-1\right)}}\end{array}$$

${M}_{between}$ refers to the mean squared deviation between units from a one-factor ANOVA, and ${M}_{within}$ refers to the mean squared deviation within units from a one-factor ANOVA. The parameter *n* denotes the number of replicate measurements for each sample (n = 3), while *N* represents the number of sites selected for the drum homogeneity study (N = 6).

### Stability study

Immediately after bottling of the selected units for the stability study they were subjected to an isochronous accelerated aging procedure, following the method described by A. Lamberty [30]. The samples were stored at 4 °C, 22 °C, and 40 °C for a period up to 6 months. After 2, 4, and 6 months, individual units were transferred from their respective storage temperatures to −18 °C. Subsequently, all units were analyzed for EAs under the repeatability conditions outlined above, alongside two reference samples that had been stored at −18 °C since bottling.

### Data analysis

MassHunter Quantitative Analysis software, Version 12.1 from Agilent was used to integrate the peak areas, to calculate the linear regressions, and to determine the analyte concentration based on SA or ISTD method.

Quantitative data were compared and visualized using R, Version 4.4.2 (R Foundation for Statistical Computing, Vienna, Austria) and chromatograms were plotted and visualized with Origin, Version 2023 (OriginLab Corporation, Northampton, MA, USA).

## Results and discussion

### HPLC-MS/MS of EAs

**Fig. 2:**
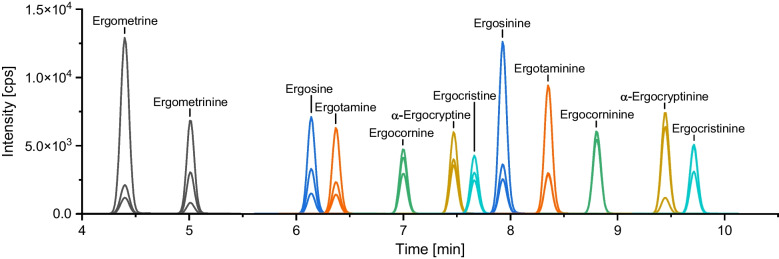
Chromatogram of a calibrator sample containing the monitored EAs at a mass concentration of 2 ng/mL. Epimers of the same EA are represented in the same color, together with their selected and optimized MRMs for the quantifier ion and two qualifier ions. For ergotaminine, ergocorninine, and ergocristinine, the distinction between the quantifier and one or more qualifier signals is not clearly distinguishable due to the similar signal intensities of the monitored transitions

The separation and quantification of EAs can be challenging. Under acidic chromatographic conditions the EAs are protonated and poor peak shapes and separation of the epimers are often observed [31]. These challenges can be overcome when working under basic conditions, leading to a better peak shape and signal-to-noise ratio. We optimized our method under basic conditions to achieve a baseline separation of all 12 priority EAs and their specific epimers in under 10 min as shown in Fig. 2. For each native and isotope-labeled epimer a multiple reaction monitoring (MRM) method for a set of one quantifier- and two qualifier ions was optimized. The optimization for each epimer was necessary as the *8R*- and *8S*-configuration showed different fragment ion patterns and intensities at discrete fragmentation energies. For the simple lysergic acid amides, ergometrine and ergometrinine, a significant difference was observed in the relative ratio between the quantifier ion (*m/z* 223) and the two most intense qualifier ions (*m/z* 207 and 208) of the native compounds compared to their ^13^CD_3_-labeled analogues within the ISTD (^13^CD_3_-qualifier *m/z* 227). Due to this discrepancy, we proposed that for the native analyte at *m/z* ratios 207 and 208, two superimposed fragment ions with the same nominal mass are present. One of these fragment ions contains the *N*^*6*^-atom and the bound methyl group, while the other one is lacking the *N*^*6*^-atom (c.f. Fig. 1). For the ISTD, these two fragment ions are no longer superimposed, as the fragment ion containing the isotope-labeled methyl group at the *N*^*6*^-atom shows a mass shift of 4 Da. This hypothesis is supported by the fragment ion spectra of the ^13^CD_3_-labeled compounds, where fragment ions with a mass shift of 4 Dalton at *m/z* 211 and 212 alongside those at *m/z* 207 and 208 were detected (Data not shown). Consequently, these two qualifier ions were unsuitable for SIDA and led to the selection of less intense but suitable qualifier ions (native *m/z* 283, 197, 180; ^13^CD_3_-labeled 283, 201, 180). The optimized MRM transitions and HPLC retention time for each of the monitored EAs are given in the SI Table S1.

LODs and LOQs for the analysis of EAs were determined using the calibration curve method in accordance with DIN 32645 and are presented in detail in Figure S1. The LODs ranged from 17 to 58 ng/kg, the LOQs from 67 to 214 ng/kg respectively and were all below the lowest calibration point of 500 ng/kg. They demonstrate that the sensitivity of the method exceeds the requirements set by European Commission Regulation 2023/915 and its suitability for the reliable assessment of food safety.

### Method comparison study

**Fig. 3:**
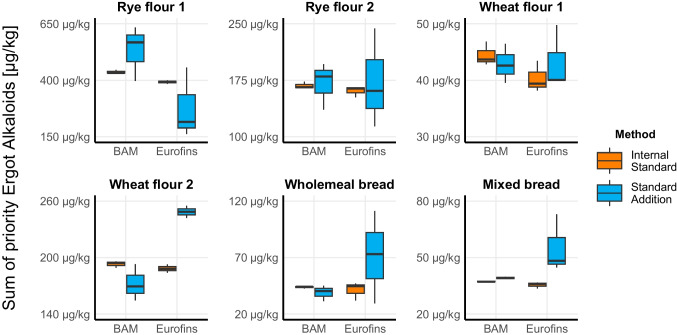
Method comparison for the sum of the 12 priority EAs in different flour and bread samples. The results obtained using the ISTD method (orange) are compared against the SA method (blue) from both laboratories BAM and Eurofins

Within this study we aimed to assess the analytical performance of the SA protocol described in EN 17425 for the quantification of EAs in different matrices compared to an SIDA method utilizing our previously synthesized ISTDs. To provide a robust performance assessment and external quality assurance, we invited Eurofins WEJ Contaminants GmbH to participate in the study as a second, independent laboratory. Eurofins provided four different flour and two different bread samples which were analyzed in triplicates with the SA and ISTD method. The individual results for each sample, method, and analyte for BAM and Eurofins are given in SI Tables S6–S29, while in Fig. 3 the results are shown for the sum of the 12 priority EAs. The level of contamination was found to vary across the investigated samples, with the highest level of EAs found in rye flour, followed by wheat flour and the bread samples showed the lowest level of EAs. This difference may be related to the thermal instability of the analytes at elevated temperatures (Fig. 7), as the flours were exposed to elevated temperatures during bread baking, which may have contributed to a reduction in detectable EAs.

A significant improvement in precision across all samples was observed in both laboratories when using the ISTD method. This effect is most evident in rye flour 1, the sample with the highest level of contamination. Therein, the ISTD method yielded comparable results between BAM and Eurofins, with measured mass fractions for the sum of the 12 EAs of 437.3 µg/kg ± 9.1 µg/kg and 392.3 µg/kg ± 13.8 µg/kg respectively. In contrast, the SA method yielded results that were not comparable with 532.9 µg/kg ± 116.1 µg/kg and 278.1 µg/kg ± 119.2 µg/kg (Table 2). For rye flour 2 and wheat flour 1, both methods yielded consistent results across both laboratories. The SA method showed a greater span width of results, which corresponds to a larger standard deviation and an overall lower precision. For wheat flour 2, wholegrain bread, and mixed bread, the ISTD results from both laboratories overlap with those obtained by BAM using the SA method. In contrast, the SA results from Eurofins differed substantially from the corresponding ISTD results, by 24.3%, 42.2%, and 36.0%, respectively.

**Table 2:** Corrected mass fraction [µg/kg] for the sum of the 12 priority EAs for the two different laboratories and methods

**Sample**	**BAM—Internal Standard** **Mass fraction ± Std. Dev. [µg/kg]**	**Eurofins—Internal Standard** **Mass fraction ± Std. Dev. [µg/kg]**
Rye flour 1	437.3 ± 9.1	392.3 ± 13.8
Rye flour 2	167.9 ± 3.8	160.7 ± 4.3
Wheat flour 1	44.5 ± 1.3	40.3 ± 1.7
Wheat flour 2	193.1 ± 3.3	188.3 ± 3.7
Wholemeal bread	43.8 ± 3.0	41.2 ± 7.5
Mixed bread	37.3 ± 0.7	35.4 ± 1.5
	**BAM—Standard Addition**	**Eurofins—Standard Addition**
Rye flour 1	532.9 ± 116.1	278.1 ± 119.2
Rye flour 2	170.8 ± 19.6	172.9 ± 38.6
Wheat flour 1	42.9 ± 2.2	43.3 ± 3.8
Wheat flour 2	172.4 ± 11.5	248.6 ± 18.2
Wholemeal bread	38.9 ± 8.2	71.3 ± 34.0
Mixed bread	39.2 ± 1.2	55.3 ± 8.7

**Fig. 4:**
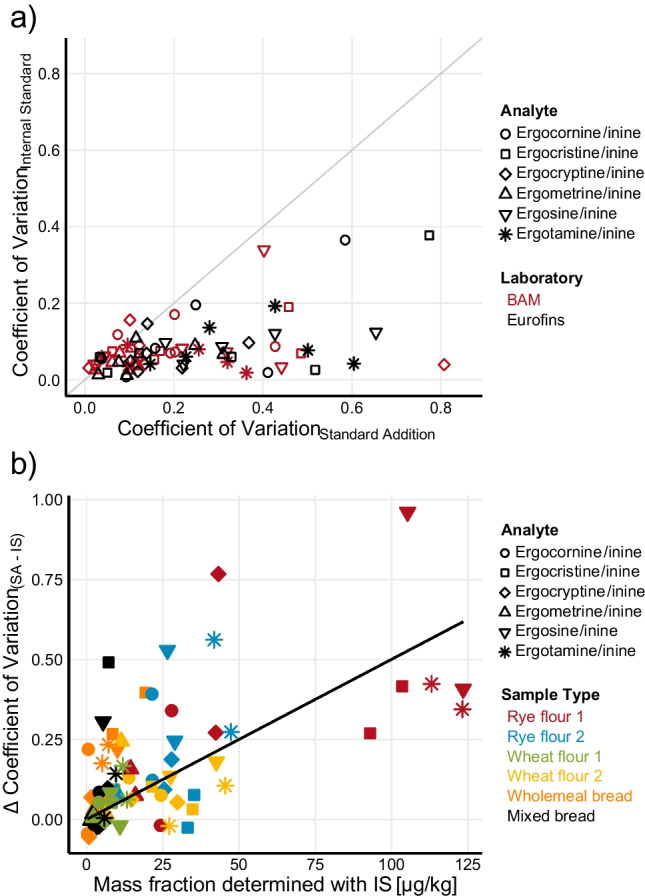
Comparison of the performance of the SA and ISTD from both laboratories: (**a**) CVs from the ISTD method plotted against CVs from the SA method (**b**) ΔCV of the two methods plotted against the determined mass fraction from the ISTD method, with a black trendline highlighting the relationship between CV and mass fraction

For an initial comparison of the two analytical methods and laboratories, we used the sum of the 12 epimers rather than each epimer or epimer pair. This approach improves clarity and simplifies the comparison by reducing the number of parameters to one per method. Because these epimers are interconvertible, the mass fraction of individual epimers may vary between laboratories due to factors such as prolonged sample preparation, light exposure, or delays before analysis, although their combined sum remains constant. Nonetheless, this simplification prevents the detection of potential trends in the assessment of EA quantification performance across the different methods. Figure 4a illustrates the coefficient of variation (CV), also referred to as relative standard deviation, for the ISTD method plotted against the corresponding CV from the SA method. Shown are the respective epimer pairs in the six samples, as analyzed by BAM (red) and Eurofins (black). Data points on the light gray line indicate equal CVs for both methods. Points above the line indicate smaller CVs for the SA method, whereas points below indicate smaller CVs for the ISTD method. Overall, the ISTD method demonstrated a significantly higher precision, with 69 of 72 (95.8%) quantifications yielding CV < 0.2, compared to only 39 of 72 (54.1%) for the SA method. The SA method performed worst for ergotamine/-inine and ergosine/-inine (8/12 results with CV > 0.2), followed by ergocryptine/-inine and ergocornine/-inine (5/12 results each).

To further investigate these, Fig. 4b presents the ΔCV (the difference between SA and ISTD CVs) plotted against the analyte mass fraction determined by the ISTD method. A ΔCV of 0 indicates comparable precision between the two methods for a given pair of epimers. The black trend line has a positive slope, indicating that the precision of the SA method described in EN 17425 decreases as the EA mass fraction increases. No significant trend appears for individual EAs across different samples, which suggests that, for the SA method, precision depends primarily on the EA mass fraction within the sample.

### Recovery rates and matrix effects

The recovery rate (RR) is an important parameter, as it directly reflects the precision and reliability of the analytical method. It indicates the proportion of analyte that has been successfully extracted, purified, and quantified from the sample. A low RR may indicate an incomplete extraction, analyte loss during purification, or signal suppression in LC-MS/MS due to coeluting matrix components. These issues can lead to inaccurate quantification by either overestimation or underestimation of the true analyte concentration. To ensure accurate results, measured concentrations are often corrected using RRs, which according to regulatory guidelines should fall within 70–120% for mycotoxins [32].

In our study, we applied the method described in EN 17425, which has been validated for flour and bread matrices and demonstrated satisfactory RRs during the method validation study. For the method comparison study, the RRs for the SA method were determined as described in Eq. 1. To assess the RRs for the ISTD method, the ratio of the peak area of the ISTD within the spiked samples and the average peak area of the ISTD in the calibration standards was calculated. The mean RRs across the six samples for both methods and laboratories are presented in Table 3.

**Table 3:** Mean recovery rates (RR) and standard deviations from the quantification of EAs in all six analyzed samples using the isotope-labeled internal standard (ISTD) and standard addition (SA) method for BAM and Eurofins

Analyte	BAM RR_ISTD_ ± Std. Dev. [%]	BAM RR_SA_ ± Std. Dev. [%]	Eurofins RR_ISTD_ ± Std. Dev. [%]	Eurofins RR_SA_ ± Std. Dev. [%]
Ergocornine	90 ± 3	86 ± 15	65 ± 6	67 ± 8
Ergocorninine	86 ± 3	88 ± 5	77 ± 15	72 ± 9
Ergocristine	95 ± 3	113 ± 23	75 ± 8	94 ± 23
Ergocristinine	105 ± 3	93 ± 11	101 ± 7	85 ± 12
α-Ergocryptine	93 ± 3	93 ± 14	59 ± 4	65 ± 9
α-Ergocryptinine	94 ± 4	92 ± 4	89 ± 9	82 ± 6
Ergometrine	95 ± 2	94 ± 5	100 ± 5	79 ± 9
Ergometrinine	104 ± 2	99 ± 2	126 ± 3	101 ± 5
Ergosine	93 ± 3	114 ± 28	87 ± 8	85 ± 46
Ergosinine	86 ± 2	84 ± 8	81 ± 10	73 ± 11
Ergotamine	92 ± 3	99 ± 18	88 ± 10	105 ± 31
Ergotaminine	96 ± 3	80 ± 9	80 ± 20	70 ± 20

Within each laboratory, the RRs for individual EAs obtained with the SA and ISTD methods were comparable. However, the ISTD method consistently shows a lower standard deviation than the SA method. This difference can be attributed to the simplified design of the SA method, which relies on a single-point addition. Furthermore, as both the unspiked and spiked samples must be extracted and analyzed consecutively, even small variations in extraction efficiency or during measurement can noticeably affect the calculated RRs. This effect becomes more prominent at higher contamination levels, when the mass fraction used for spiking (EN 17425; 10 µg/kg) is low compared with the native contamination level (e.g., rye flour 1: ergotamine 97.8 ± 2.0 µg/kg, BAM; 94.3 ± 8.7 µg/kg, Eurofins, determined with ISTD).

The interlaboratory comparison showed greater variability. At BAM, mean RRs for all EAs were within the target range of 70–120%, except for ergocornine with the SA method. In contrast, Eurofins reported low RRs for ergocornine and α-ergocryptine for both methods (Table 3). Since all ergopeptines are structurally similar, it was unexpected that only 2 of the 10 analytes consistently showed reduced RRs. The standard deviations for these compounds were not increased, suggesting only minor variability across the different samples.

However, this finding resembled observations during method development at BAM, where ergocristine and ergosinine showed low average RRs. Those results were obtained on a Phenyl-Hexyl column, commonly used for EA quantification [33, 34]. To test whether this effect was caused by extraction inefficiency or matrix interference, a flour sample was split into six aliquots: three were spiked with ISTD before extraction and cleanup and three after. Both sets yielded similarly low RRs for ergocristine and ergosinine, pointing to matrix effects rather than a loss during extraction. Further attempts to optimize the chromatographic separation on the Phenyl-Hexyl column were not successful.

**Fig. 5:**
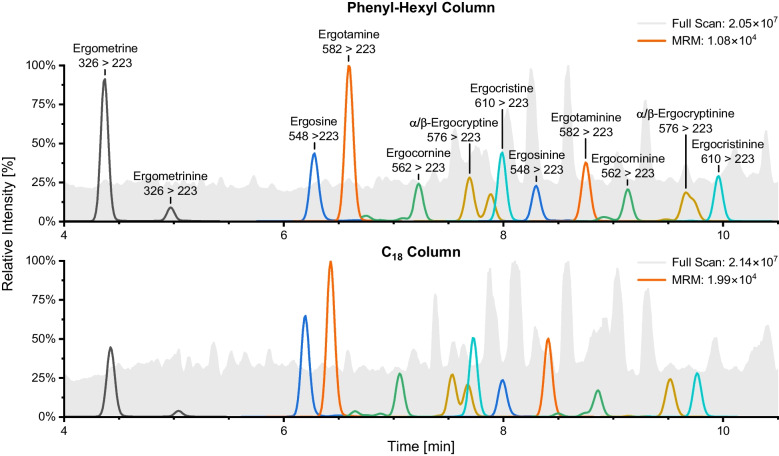
Chromatograms for the separation of EAs in the same rye flour sample, measured consecutively on a Phenyl-Hexyl (150 × 2 mm, 3 µm) and C_18_ (150 × 2.1, 1.7 µm) column. The MRM transitions [M+H]^+^ are shown in color; full scan (*m/z* 300–1000) in gray. Intensities are normalized to the respective base peak

To evaluate whether changing the stationary phase would influence this effect, the analysis was repeated on a C_18_ column, as the column chemistry can significantly influence the retention behavior of both target analytes and co-eluting matrix constituents. Figure 5 shows the normalized MRM chromatograms obtained from both columns, overlaid with a normalized full scan (m/z 300–1000) for a visualization and comparison of eluting matrix components.

The full scan chromatogram from the Phenyl-Hexyl column reveals pronounced matrix co-elution at the retention times of ergocristine (7.99 min) and ergosinine (8.30 min). The ergocristine peak overlaps partially with a matrix signal, while the ergosinine peak coincides fully with a major matrix component. This results in average RRs of 72% and 32%, respectively. On the C_18_ column, ergocristine eluted at 7.73 min without co-elution of major matrix components, whereas ergosinine eluted at 8.00 min in a valley between two pronounced matrix peaks. Under these chromatographic conditions RRs of 97% for ergocristine and 92% for ergosinine were achieved during method development.

Although the RRs for ergocornine and α-ergocryptine obtained by Eurofins were below the commonly accepted threshold of 70%, the use of an ISTD effectively corrected for matrix effects, enabling comparable quantification results between BAM and Eurofins.

### Rye flour reference material

For the production of the rye flour RM, 19 samples from 10 different manufacturers were purchased and the total EA content was analyzed (Table S2). For one manufacturer, the EA content in four different flours from a single batch ranged from 469 µg/kg up to 934 µg/kg, with two out of four samples exceeding the regulatory limits. Although such highly contaminated samples should not be sold to end consumers, sourcing naturally contaminated samples with sufficiently high levels for RM production can be challenging. Spiking the RM with the analyte, here ground sclerotia containing EA, remains only an alternative, as factors like particle size or absorption behavior on the matrix often differ from those of naturally contaminated samples. Highly contaminated samples were blended with less contaminated ones and sufficiently homogenized to obtain a RM with an EA mass fraction between 250 and 300 µg/kg.

The characterization of the rye flour RM comprised the evaluation of the drum homogeneity and stability to assign mass fraction values for each of the limit-relevant priority EA based on ISO 33405. Furthermore, the uncertainty budget for each EA was calculated to support the RM based upon the primary ratio method of SIDA-HPLC-ESI-MS/MS.

### Assessment of homogeneity

The homogeneity study estimates how consistently the analytes are distributed throughout the RM batch. This is achieved through the statistical comparison of the variation observed among different units within a batch with the inherent precision of the method. The between-unit standard deviation is determined from this comparison and serves as the basis for estimating the uncertainty due to potential heterogeneity.

**Fig. 6:**
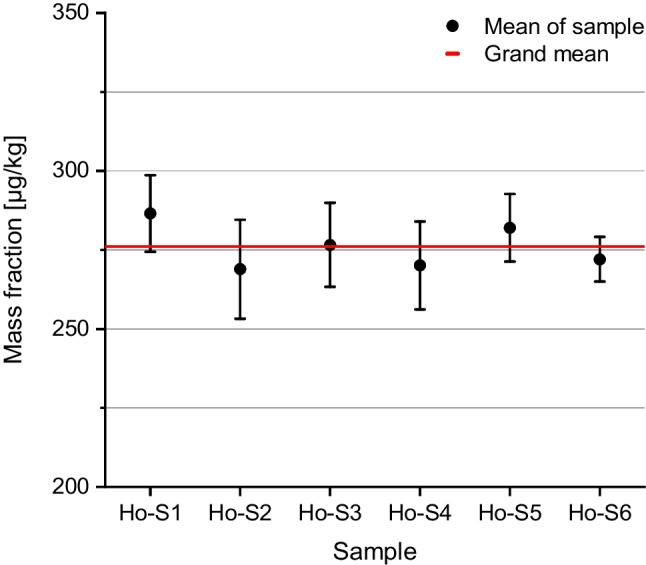
Homogeneity study of the rye flour RM for the sum of 12 monitored EAs. The figure displays the mean mass fractions from six samples taken at different positions after homogenization. Each sample was extracted and analyzed in triplicate (n = 3), with error bars representing the standard deviations. The grand mean concentration (red line) is highlighted at 276.0 µg/kg

Given the fine particle size of the flour and the thorough homogenization of the batch using a drum hoop mixer, a high level of homogeneity was anticipated. Therefore, six samples were taken from different positions within the drum and each sample was extracted and analyzed in triplicate in a randomized order. The results for the sum of the 12 monitored EAs are presented in Fig. 6.

The results in Fig. 6 indicate no observable trend related to the sampling position within the drum. Results for the individual EAs were assessed using single-factorial ANOVA (Table S5). Since the P-values for all EAs exceeded the significance level (α = 0.05), the ANOVA null hypothesis could not be rejected. This suggests that, for a sample size of 10 g, the material can be considered sufficiently homogeneous. The between-unit homogeneity (*s*_*bu*_) was calculated in accordance with Eq. 4. In cases where *M*_*between*_ < *M*_*within*_ the Eq. 4 is inapplicable, ISO 33405 permits to set *s*_*bu*_ to zero and estimate a maximum between-unit variability via Eq. 5. In line with the recommendations of Linsinger et al*.*, and adopting a conservative approach, both relative values *u*_*bu,r*_ and *u*_*bu,min,*r_ were calculated whenever feasible [35]. The larger of the two was then used to account for the contribution of inhomogeneity (*u*_*bu*_) in the calculation of the overall uncertainty budget of the RM.

### Assessment of stability

The isochronous stability study was conducted to assess the temperature stability of the analytes within its matrix. For this purpose, multiple samples of the RM were stored at 4 °C, 22 °C, or 40 °C for up to 6 months. At 2-month intervals, samples were transferred to the reference temperature (− 18 °C). After 6 months, all samples were extracted and analyzed in a randomized sequence to minimize bias (Table S3).

**Fig. 7:**
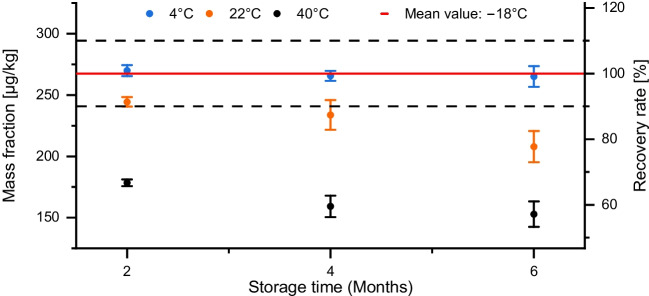
Results of the isochronous stability study showing the sum of the 12 monitored EAs stored at different temperatures over a 6-month period. The red line represents the mean value of two reference samples stored at − 18 °C, which were extracted and analyzed in triplicate (n = 6). The dashed lines indicate the acceptance criteria of ± 10%. Samples stored at elevated temperatures (4°C, 22 °C, and 40 °C) were extracted and analyzed in triplicate (n = 3), with error bars representing the calculated standard deviations

The results are shown in Fig. 7 and indicate that the sum of the 12 investigated EAs remained stable at 4 °C and −18 °C throughout the 6-month observation period. Furthermore, no changes in the epimer ratios were observed between the homogeneity and stability study (c.f. Table 4 and Table S5). At temperatures of 22 °C and 40 °C, the EA content in the material decreased significantly by 23% and 38% over 6 months, respectively.

**Table 4:** Mass fraction for the analyzed EAs within the RM and their expanded uncertainties

Ergot alkaloid	Mass fraction [µg/kg]	Expanded uncertainty [µg/kg]
Ergocornine	29.8	3.6
Ergocorninine	18.1	2.0
Ergocristine	42.9	5.0
Ergocristinine	14.5	1.6
α/β-Ergocryptine	34.3	3.8
α/β-Ergocryptinine	15.7	1.7
Ergometrine	9.8	1.1
Ergometrinine	1.6	0.2
Ergosine	27.1	3.1
Ergosinine	13.0	1.4
Ergotamine	45.5	5.8
Ergotaminine	15.1	2.0
**∑ 12 priority EAs**	**267.5**	**10.6**

Based on the specific mass fractions determined in the homogeneity and stability study, the observed changes over a period of 6 months for the EAs were negligible compared to the overall RM uncertainty when stored at a temperature of 4 °C or below. Consequently, the uncertainty due to long-term stability was set to zero, in accordance with ISO 33405.

### Assessment of uncertainty

The relative combined uncertainty (*u*_*com,r*_) of the RM was composed from the relative uncertainty contributions of the homogeneity study (*u*_*bu,r*_), stability study (*u*_*stab,r*_; was set to zero), and the EA calibration standards (Table S4) according to its certificate (*u*_*pur,r*_) and from sample handling (*u*_*hand,r*_—pragmatic approach 5% for all EAs) using Eq. 6.

6 $$\begin{array}{c}{u}_{com,r}=\sqrt{{u}_{bu,r}^{2}+{u}_{stab,r}^{2}+{u}_{pur,r}^{2}+{u}_{hand,r}^{2}}\end{array}$$

Expanded uncertainties (*U*) of the EA values were calculated by applying a coverage factor (*k*) of *k* = 2 (Eq. 7), which corresponds to a coverage probability of about 95%.

7 $$\begin{array}{c}U=k\times {u}_{com,r}\end{array}$$

The mass fractions of the individual EAs from the stability study, together with their total sum and expanded uncertainties, are given in Table 4.

The expanded uncertainties for the individual EAs range from 11 to 13%. For the sum of the 12 EAs, the combined uncertainty, calculated using Gaussian error propagation, is *U* = 4% and lower than the uncertainties of the individual EAs. The reduction occurs because errors from different independent measurements can offset each other, resulting in a combined uncertainty smaller than the linear sum of the individual errors. Furthermore, these excellent results highlight the precision of the method and emphasize its contribution to the production of RMs.

## Conclusion

Routine monitoring of EAs in grain-based food products is necessary to comply with EU legal requirements and to ensure consumer safety. Therefore, we compared a SA against an ISTD approach, employing our self-developed ISTDs. The SA method prescribed in EN 17425 achieved acceptable results within the performed interlaboratory comparison studies for concentrations up to approximately 175 µg/kg. A notable decline in the precision of the SA method was observed at elevated contamination levels. Additionally, the results obtained from BAM and Eurofins were not consistently comparable, even at lower contamination levels. This poses a particular concern for infant and young children’s formula, where EU regulatory limits require EA levels below 20 µg/kg. These findings are consistent with an EN 17425 validation study, which reported satisfactory performance only within the range of 24.1 to 168 µg/kg [17].

Considering these limitations, the current EN 17425 method is insufficient for reliable quantification across all regulated levels. Potential improvements to the present methodology may include increasing the amount of standard used for the single SA approach or implementing a multi-level SA strategy. However, both modifications would lead to increased costs and workload due to higher consumption of standards and the necessity of additional time for sample preparation and measurement.

Our interlaboratory comparison study demonstrated that the adoption towards an ISTD approach, without altering any other EN 17425 parameters, significantly enhances both precision and trueness. In collaboration with Eurofins, we confirmed that the ISTD approach yields interlaboratory comparable results, even at higher contamination levels. Furthermore, the ISTD approach has been demonstrated to provide enhanced compensation for matrix effects and to reduce the analysis time per sample, given that only a single extraction and measurement is required per sample.

To further support method validation and routine testing, we produced a RM for the 12 priority EAs in rye flour. Homogeneity and long-term stability of the RM were evaluated based on ISO 33405, demonstrating sufficient stability for at least 6 months when storing the RM at 4 °C or below.

These findings highlight the potential of ISTD-based quantification to improve the reliability and comparability of EA analysis by HPLC-MS/MS. Given the analytical challenges posed by climate change, developments in the production of highly processed foods, and the emergence of grain-based meat substitutes, these ISTD can provide a robust solution for ensuring analytical reliability and safeguarding consumer safety.

## Supplementary Information

Below is the link to the electronic supplementary material.

Supplementary file1 (DOCX 122 KB)

## Data Availability

Data are contained within the article and supplementary materials.

## References

[1] Flieger M, Wurst M, Shelby R. Ergot alkaloids–sources, structures and analytical methods. Folia Microbiol (Praha). 1997;42:3–29. 10.1007/BF02898641. 9160999 10.1007/BF02898641

[2] Scott PM. Analysis of ergot alkaloids - a review. Mycotoxin Res. 2007;23:113–21. 10.1007/BF02951506. 23605988 10.1007/BF02951506

[3] Pitt JI, Miller JD. A Concise History of Mycotoxin Research. J Agric Food Chem. 2017;65:7021–33. 10.1021/acs.jafc.6b04494. 27960261 10.1021/acs.jafc.6b04494

[4] Keller NP, Turner G, Bennett JW. Fungal secondary metabolism - from biochemistry to genomics. Nat Rev Microbiol. 2005;3:937–47. 10.1038/nrmicro1286. 16322742 10.1038/nrmicro1286

[5] Miedaner T, Geiger HH. Biology, genetics, and management of ergot (*Claviceps* spp.) in rye, sorghum, and pearl millet. Toxins (Basel). 2015;7:659–78. 10.3390/toxins7030659. 25723323 10.3390/toxins7030659 PMC4379517

[6] Young JC, Chen Z-j, Marquardt RR. Reduction in alkaloid content of ergot sclerotia by chemical and physical treatment. J Agric Food Chem. 1983. 10.1021/jf00116a057. 6853863 10.1021/jf00116a057

[7] Fajardo JE, Dexter JE, Roscoe MM, Nowicki TW. Retention of ergot alkaloids in wheat during processing. Cereal Chem. 1995;72:291–8.

[8] Crews C. Analysis of Ergot Alkaloids. Toxins (Basel). 2015;7:2024–50. 10.3390/toxins7062024. 26046699 10.3390/toxins7062024 PMC4488688

[9] Jakubczyk D, Cheng JZ, O’Connor SE. Biosynthesis of the ergot alkaloids. Nat Prod Rep. 2014;31:1328–38. 10.1039/c4np00062e. 25164781 10.1039/c4np00062e

[10] Cherewyk JE, Blakley BR, Al-Dissi AN. The C-8-S-isomers of ergot alkaloids - a review of biological and analytical aspects. Mycotoxin Res. 2024;40:1–17. 10.1007/s12550-023-00507-0. 37953416 10.1007/s12550-023-00507-0 PMC10834577

[11] Volnin A, Parshikov A, Tsybulko N, Mizina P, Sidelnikov N. Ergot alkaloid control in biotechnological processes and pharmaceuticals (a mini review). Front Toxicol. 2024;6:1463758. 10.3389/ftox.2024.1463758. 39439532 10.3389/ftox.2024.1463758 PMC11493748

[12] EFSA. Scientific opinion on ergot alkaloids in food and feed. EFSA J. 2012. 10.2903/j.efsa.2012.2798. 10.2903/j.efsa.2012.2798 PMC13093078 42016129

[13] Krska R, Crews C. Significance, chemistry and determination of ergot alkaloids: a review. Food Addit Contam Part A Chem Anal Control Expo Risk Assess. 2008;25:722–31. 10.1080/02652030701765756. 18484300 10.1080/02652030701765756

[14] Commission Regulation (EC) No 2021/1399 of 24 August 2021 amending Regulation (EC) No 1881/2006 as regards maximum levels of ergot sclerotia and ergot alkaloids in certain foodstuffs. Off J Eur Union. 2021;L301:1–5. Available from: https://eur-lex.europa.eu/eli/reg/2021/1399/oj/eng.

[15] Commission Regulation (EU) 2023/915 of 31 March 2023 amending Regulation (EC) No 1881/2006 as regards maximum levels of certain contaminants in foodstuffs. Off J Eur Union. 2023;L119:103–157. Available from: https://eur-lex.europa.eu/eli/reg/2023/915/oj/eng.

[16] Commission Regulation (EU) 2024/1808 of 1 July 2024 amending Regulation (EU) 2023/915 as regards the application date of lower maximum levels for ergot sclerotia and ergot alkaloids in food. Off J Eur Union. 2024;OJ L, 2.7.2024: (2024/1808):1–3. Available from: https://eur-lex.europa.eu/eli/reg/2024/1808/oj/eng.

[17] European Committee for Standardization (CEN). Technical Committee 275. Foodstuffs — determination of ergot alkaloids in cereals and cereal products by dSPE clean‑up and HPLC‑MS/MS; EN 17425:2021. 2021;44. Available from: https://www.dinmedia.de/de/norm/din-en-17425/326671005.

[18] Steiner D, Humpel A, Stamminger E, Schoeberl A, Pachschwoell G, Sloboda A, et al. An interlaboratory comparison study of regulated and emerging mycotoxins using liquid chromatography mass spectrometry: Challenges and future directions of routine multi-mycotoxin analysis including emerging mycotoxins. Toxins (Basel). 2022. 10.3390/toxins14060405. 35737066 10.3390/toxins14060405 PMC9229327

[19] Steiner D, Malachova A, Sulyok M, Krska R. Challenges and future directions in LC-MS-based multiclass method development for the quantification of food contaminants. Anal Bioanal Chem. 2021;413:25–34. 10.1007/s00216-020-03015-7. 33188454 10.1007/s00216-020-03015-7 PMC7801304

[20] Varga E, Glauner T, Berthiller F, Krska R, Schuhmacher R, Sulyok M. Development and validation of a (semi-)quantitative UHPLC-MS/MS method for the determination of 191 mycotoxins and other fungal metabolites in almonds, hazelnuts, peanuts and pistachios. Anal Bioanal Chem. 2013;405:5087–104. 10.1007/s00216-013-6831-3. 23471368 10.1007/s00216-013-6831-3 PMC3656230

[21] Kleigrewe K, Niehaus EM, Wiemann P, Tudzynski B, Humpf HU. New approach via gene knockout and single-step chemical reaction for the synthesis of isotopically labeled fusarin c as an internal standard for the analysis of this fusarium mycotoxin in food and feed samples. J Agric Food Chem. 2012;60:8350–5. 10.1021/jf302534x. 22877497 10.1021/jf302534x

[22] Varga E, Glauner T, Koppen R, Mayer K, Sulyok M, Schuhmacher R, et al. Stable isotope dilution assay for the accurate determination of mycotoxins in maize by UHPLC-MS/MS. Anal Bioanal Chem. 2012;402:2675–86. 10.1007/s00216-012-5757-5. 22293971 10.1007/s00216-012-5757-5 PMC3292730

[23] Mandal P, Chakraborty S, Bera R, Karmakar S, Pal TK. Internal standard an important analyte use in drug analysis by liquid chromatography mass spectrometry- an article. Int J Pharm Biomed Sci. 2022;02:10–7. 10.47191/ijpbms/v2-i1-02.

[24] Rychlik M, Asam S. Stable isotope dilution assays in mycotoxin analysis. Anal Bioanal Chem. 2008;390:617–28. 10.1007/s00216-007-1717-x. 18060393 10.1007/s00216-007-1717-x

[25] Li D, Steimling JA, Konschnik JD, Grossman SL, Kahler TW. Quantitation of mycotoxins in four food matrices comparing stable isotope dilution assay (SIDA) with matrix-matched calibration methods by LC–MS/MS. J AOAC Int. 2019;102:1673–80. 10.5740/jaoacint.19-0028. 30940286 10.5740/jaoacint.19-0028

[26] Berglund M. Chapter 37 - Introduction to Isotope Dilution Mass Spectrometry (IDMS). In: de Groot PA, editor. Handbook of Stable Isotope Analytical Techniques. Amsterdam: Elsevier; 2004. p. 820–34.

[27] Herter SO, Haase H, Koch M. First synthesis of ergotamine-(13)CD(3) and ergotaminine-(13)CD(3) from unlabeled ergotamine. Toxins (Basel). 2024. 10.3390/toxins16040199. 38668624 10.3390/toxins16040199 PMC11053779

[28] Herter SO, Haase H, Koch M. Semisynthesis of stable isotope-labeled ergot alkaloids for HPLC-MS/MS analysis. J Agric Food Chem. 2025;73:18412–9. 10.1021/acs.jafc.5c03345. 40643980 10.1021/acs.jafc.5c03345 PMC12291449

[29] International Organization for Standardization. Reference materials - Approaches for characterization and assessment of homogeneity and stability ISO 33405:2024. Geneva: International Organization for Standardization; 2024.

[30] Lamberty A, Schimmel H, Pauwels J. The study of the stability of reference materials by isochronous measurements. Fresenius J Anal Chem. 1998;360:359–61. 10.1007/s002160050711.

[31] Rollo E, Catellani D, Dall’Asta C, Dreolin N, Suman M. Ergot alkaloids: comparison of extraction efficiencies for their monitoring in several cereal-solvent combinations by UPLC-MS/MS. Mycotoxin Res. 2025;41:127–46. 10.1007/s12550-024-00569-8. 39527231 10.1007/s12550-024-00569-8

[32] European Commission. Commission Implementing Regulation (EU) 2023/2782 of 14 December 2023 laying down the methods of sampling and analysis for the control of the levels of mycotoxins in food and repealing Regulation (EC) No 401/2006. Off J Eur Union. 2023;OJ L 2782:1–44. Available from: https://eur-lex.europa.eu/eli/reg_impl/2023/2782/oj?uri=CELEX:32023R2782.

[33] Lattanzio VMT, Verdini E, Sdogati S, Caporali A, Ciasca B, Pecorelli I. Undertaking a new regulatory challenge: monitoring of ergot alkaloids in Italian food commodities. Toxins (Basel). 2021. 10.3390/toxins13120871. 34941709 10.3390/toxins13120871 PMC8708126

[34] Lehner AF, Craig M, Fannin N, Bush L, Tobin T. Electrospray[+] tandem quadrupole mass spectrometry in the elucidation of ergot alkaloids chromatographed by HPLC: screening of grass or forage samples for novel toxic compounds. J Mass Spectrom. 2005;40:1484–502. 10.1002/jms.933. 16278935 10.1002/jms.933

[35] Linsinger TPJ, Pauwels J, van der Veen AMH, Schimmel H, Lamberty A. Homogeneity and stability of reference materials. Accredit Qual Assur. 2001;6:20–5. 10.1007/s007690000261.

