# Supplementary material for: Isotope dilution LC-MS/MS for the quantification of ergot alkaloids: a comparative study
Source: Anal Bioanal Chem. 2026 Jun 2;418(15):4865–78. doi: 10.1007/s00216-026-06581-4 (PMC13388356; doi:10.1007/s00216-026-06581-4)
Supplement: Supplementary file 1 — Supplementary file1 (DOCX 122 KB) [file 216_2026_6581_MOESM1_ESM.docx]

**Supplementary Information**

**Isotope-Dilution LC-MS/MS for the Quantification of Ergot Alkaloids: A Comparative Study**

Sven-Oliver Herter^a^, Susanne Krentscher^a^, Kevin Sassin^b^, Christian Kornrumpf^b^, Sarah Kulas^b^, Hajo Haase^c^ and Matthias Koch^a,*^

^a^ Bundesanstalt für Materialforschung und -prüfung (BAM), Division 1.7 Organic Trace and Food Analysis, Richard‑Willstätter-Str. 11, 12489 Berlin, Germany

^b^ Eurofins WEJ Contaminants GmbH, Neuländer Kamp 1, 21079 Hamburg

^c^ Technische Universität Berlin, Department of Food Chemistry and Toxicology, Gustav-Meyer-Allee 25, 13355 Berlin, Germany

^*^ Corresponding author: [matthias.koch@bam.de](mailto:matthias.koch@bam.de)

**Table S1** Chromatographic retention times, multiple reaction monitoring (MRM) transitions, and optimized collision energies for the quantification of ^13^CD_3_-labeled and native ergot alkaloids

|  | **Retention Time [min]** | ***m/z* Precursor ion-^13^CD_3_ (native) [M+H]^+^** | **Quantifier-^13^CD_3_ (native) Qualifier 1-^13^CD_3_ (native) Qualifier 2-^13^CD_3_ (native)** | **Collision Energy [V]** |
| --- | --- | --- | --- | --- |
| Ergometrine | 4.40 | 330.2 (326.2) | 227 (223) 283 (283) 201 (197) | 28 24 28 |
| Ergometrinine | 5.00 | 330.2 (326.2) | 227 (223) 283 (283) 180 (180) | 28 24 48 |
| Ergosine | 6.14 | 552.3 (548.3) | 227 (223) 277 (277) 272 (268) | 40 28 28 |
| Ergotamine | 6.37 | 586.3 (582.3) | 227 (223) 277 (277) 272 (268) | 36 28 23 |
| Ergocornine | 7.00 | 566.3 (562.3) | 227 (223) 305 (305) 272 (268) | 40 28 28 |
| α-Ergocryptine | 7.47 | 580.3 (576.3) | 227 (223) 305 (305) 272 (268) | 40 28 28 |
| Ergocristine | 7.66 | 614.3 (610.3) | 227 (223) 305 (305) 272 (268) | 40 32 28 |
| Ergosinine | 7.92 | 552.3 (548.3) | 227 (223) 277 (277) 263 (263) | 36 28 28 |
| Ergotaminine | 8.35 | 586.3 (582.3) | 227 (223) 297 (297) 277 (277) | 36 32 32 |
| Ergocorninine | 8.80 | 566.3 (562.3) | 227 (223) 305 (305) 277 (277) | 40 28 32 |
| α-Ergocryptinine | 9.44 | 580.3 (576.3) | 227 (223) 305 (305) 272 (268) | 40 32 32 |
| Ergocristinine | 9.72 | 614.3 (610.3) | 227 (223) 305 (305) 325 (325) | 40 28 28 |

**Figure S1** Results for the determination of the limit of detection (LOD) and limit of quantification (LOQ) for each of the analyzed EA

**Table S2** Measured mass fraction for the sum of the twelve priority EAs from the screening of 19 different rye flours from local German supermarkets for the reference material production

| **Rye flour Sample** | **Mass fraction [µg/kg] ± Std. Dev. [µg/kg]** |
| --- | --- |
| A01 | 19.8 ± 3.7 |
| A02 | 934.1 ± 17.6 |
| A02-1 | 477.8 ± 10.9 |
| A02-2 | 469.2 ± 9.8 |
| A02-3 | 650.5 ± 12.8 |
| B01 | 1.2 ± 0.8 |
| B01-1 | <LOQ |
| C01 | 67.5 ± 2.9 |
| C01-1 | 62.1 ± 1.8 |
| C01-2 | 59.1 ± 3.6 |
| C02 | 8.2 ± 0.6 |
| D01 | <LOQ |
| D01-1 | 4.9 ±0.8 |
| D02 | 29.3 ± 1.9 |
| D02-1 | 33.0 ± 1.4 |
| D03 | 110.3 ± 26.2 |
| D04 | 25.2 ± 2.9 |
| D04-1 | 18.6 ± 1.8 |
| D05 | 8.7 ± 1.2 |
| <LOQ – all EAs below 0.5 µg/kg | |

**Table S3** Results for the mass fraction of the twelve priority EAs from the 6-month stability study at different temperatures and storage durations

| **Temperature** | **2 Months** | **4 Months** | **6 Months** | |
| --- | --- | --- | --- | --- |
| −18 °C | *n.a.* | *n.a.* | 267.4 ± 4.0 µg/kg | |
| 4 °C | 269.9 ± 4.5 µg/kg | 267.7 ± 4.1 µg/kg | 265.1 ± 8.4 µg/kg | |
| 22 °C | 244.4 ± 3.9 µg/kg | 233.7 ± 12.1 µg/kg | 207.9 ± 12.8 µg/kg | |
| 40 °C | 178.4 ± 2.7 µg/kg | 159.2 ± 8.8 µg/kg | 152.8 ± 10.4 µg/kg | |
| *n.a.* − not analyzed | | | | |

**Table S4** Concentration and uncertainty of the certified standards (RomerLabs, Tulln, Austria) used for the quantification of EAs

| **Ergot alkaloid** | **Concentration [µg/mL]** | **Uncertainty *u_pur_* [µg/mL]** | **Uncertainty *u_pur,r_*** |
| --- | --- | --- | --- |
| Ergocornine | 100.8 | 1.4 | 0.0139 |
| Ergocorninine | 25.3 | 0.4 | 0.0158 |
| Ergocristine | 100.1 | 1.4 | 0.0140 |
| Ergocristinine | 25.3 | 0.4 | 0.0158 |
| α-Ergocryptine | 100.9 | 1.4 | 0.0138 |
| α-Ergocryptinine | 25.1 | 0.4 | 0.0159 |
| Ergometrine | 100.0 | 1.4 | 0.0140 |
| Ergometrinine | 25.2 | 0.4 | 0.0159 |
| Ergosine | 100.6 | 1.4 | 0.0139 |
| Ergosinine | 25.0 | 0.4 | 0.0160 |
| Ergotamine | 100.4 | 1.4 | 0.0139 |
| Ergotaminine | 25.0 | 0.4 | 0.0160 |

**Table S5** Results of the homogeneity study for the RM, based on a one-way ANOVA. Given are the values per analyte for the grand mean, *n* the number of replicates of each taken sample, *N* the number of taken samples for the homogeneity study, M_between_ the mean squared deviation between units, M_within_ the mean squared deviation within units, P-value of ANOVA, s_bb,r_ standard uncertainty between units according to Equation 4 divided be the grand mean, and s_bu,min,r_ standard uncertainty between units according to Equation 5 divided be the grand mean.

| **Ergotalkaloid** | **Grand Mean [µg/kg]** | **n** | **N** | **M_between_ [µg^2^/kg^2^]** | **M_within_ [µg^2^/kg^2^]** | **P** | **u_bu,r_** | **u_bu,min,r_** |
| --- | --- | --- | --- | --- | --- | --- | --- | --- |
| Ergocornine | 30.8 | 3 | 6 | 4.2903 | 8.0836 | 0.7493 | 0 | **0.0294** |
| Ergocorninine | 16.3 | 3 | 6 | 0.1659 | 0.5750 | 0.9103 | 0 | **0.0149** |
| Ergocristine | 44.9 | 3 | 6 | 8.5528 | 14.1448 | 0.6981 | 0 | **0.0267** |
| Ergocristinine | 15.6 | 3 | 6 | 0.4021 | 0.6224 | 0.6698 | 0 | **0.0162** |
| α/β-Ergocryptine | 34.5 | 3 | 6 | 3.7610 | 4.3999 | 0.5377 | 0 | **0.0194** |
| α/β-Ergocryptinine | 16.3 | 3 | 6 | 0.1482 | 0.3533 | 0.8264 | 0 | **0.0116** |
| Ergometrine | 10.7 | 3 | 6 | 0.3004 | 0.3906 | 0.5897 | 0 | **0.0187** |
| Ergometrinine | 2.0 | 3 | 6 | 0.0057 | 0.0072 | 0.5717 | 0 | **0.0137** |
| Ergosine | 29.1 | 3 | 6 | 0.4684 | 4.1810 | 0.9874 | 0 | **0.0225** |
| Ergosinine | 12.4 | 3 | 6 | 0.0820 | 0.4561 | 0.9649 | 0 | **0.0174** |
| Ergotamine | 48.3 | 3 | 6 | 21.0995 | 11.4829 | 0.1798 | **0.0370** | 0.0224 |
| Ergotaminine | 15.1 | 3 | 6 | 1.8096 | 0.6418 | 0.0656 | **0.0413** | 0.0170 |

# **Quantification results BAM**

**Table S6** Results for the quantification of the twelve priority ergot alkaloids in rye flour 1 using the internal standard method

| **Sample** | **Laboratory - Method** | **Analyte** | **Mass fraction [µg/kg]** | | | **Avg. Mass fraction [µg/kg]** | **Std. Dev. [µg/kg]** | **Rel. Std. Dev.** | **Avg. Recovery Rate ± Std. Dev** |
| --- | --- | --- | --- | --- | --- | --- | --- | --- | --- |
|  |  |  | **1^st^ triplicate** | **2^nd^ triplicate** | **3^rd^ triplicate** |  |  |  |  |
| Rye flour 1 | BAM - IS | Ergocornine | 18.9 | 17.2 | 20.6 | **18.9** | 1.7 | 9 % | 93 % ± 5 % |
|  |  | Ergocorninine | 9.9 | 8.0 | 9.1 | **9.0** | 1.0 | 11 % | 89 % ± 5 % |
|  |  | Ergocristine | 80.7 | 75.1 | 85.6 | **80.5** | 5.3 | 7 % | 99 % ± 5 % |
|  |  | Ergocristinine | 23.1 | 21.1 | 24.8 | **23.0** | 1.9 | 8 % | 115 % ± 9 % |
|  |  | α/β-Ergocryptine | 30.8 | 32.5 | 34.1 | **32.5** | 1.7 | 5 % | 96 % ± 5 % |
|  |  | α/β-Ergocryptinine | 10.9 | 10.3 | 10.9 | **10.7** | 0.3 | 3 % | 98 % ± 6 % |
|  |  | Ergometrine | 13.7 | 14.3 | 14.3 | **14.1** | 0.3 | 2 % | 99 % ± 4 % |
|  |  | Ergometrinine | 1.8 | 1.9 | 2.0 | **1.9** | 0.1 | 4 % | 108 % ± 6 % |
|  |  | Ergosine | 88.6 | 94.2 | 88.5 | **90.4** | 3.3 | 4 % | 92 % ± 5 % |
|  |  | Ergosinine | 33.5 | 33.9 | 31.7 | **33.0** | 1.2 | 4 % | 88 % ± 3 % |
|  |  | Ergotamine | 95.5 | 98.5 | 99.4 | **97.8** | 2.0 | 2 % | 91 % ± 4 % |
|  |  | Ergotaminine | 25.1 | 25.6 | 25.5 | **25.4** | 0.3 | 1 % | 105 % ± 8 % |

**Table S7** Results for the quantification of the twelve priority ergot alkaloids in rye flour 1 using the standard addition method

| **Sample** | **Laboratory - Method** | **Analyte** | **Mass fraction [µg/kg]** | | | **Avg. Mass fraction [µg/kg]** | **Std. Dev. [µg/kg]** | **Rel. Std. Dev.** | **Avg. Recovery Rate ± Std. Dev** |
| --- | --- | --- | --- | --- | --- | --- | --- | --- | --- |
|  |  |  | **1^st^ triplicate** | **2^nd^ triplicate** | **3^rd^ triplicate** |  |  |  |  |
| Rye flour 1 | BAM - SA | Ergocornine | 29.0 | 12.0 | 39.9 | **27.0** | 14.0 | 52 % | 78 % ± 44 % |
|  |  | Ergocorninine | 14.8 | 7.7 | 10.8 | **11.1** | 3.6 | 32 % | 74 % ± 14 % |
|  |  | Ergocristine | 97.6 | 31.8 | 68.4 | **65.9** | 33.0 | 50 % | 145 % ± 72 % |
|  |  | Ergocristinine | 53.7 | 21.1 | 31.4 | **35.4** | 16.7 | 47 % | 82 % ± 28 % |
|  |  | α/β-Ergocryptine | 32.0 | 20.0 | 122.4 | **58.1** | 56.0 | 96 % | 92 % ± 63 % |
|  |  | α/β-Ergocryptinine | 13.1 | 10.5 | 12.8 | **12.1** | 1.4 | 12 % | 85 % ± 8 % |
|  |  | Ergometrine | 14.4 | 12.2 | 11.7 | **12.8** | 1.4 | 11 % | 108 % ± 10 % |
|  |  | Ergometrinine | 2.1 | 2.1 | 2.0 | **2.1** | 0.0 | 2 % | 99 % ± 1 % |
|  |  | Ergosine | 36.9 | 105.0 | 49.1 | **63.7** | 36.3 | 57 % | 160 % ± 74 % |
|  |  | Ergosinine | 43.1 | 56.8 | 30.6 | **43.5** | 13.1 | 30 % | 70 % ± 18 % |
|  |  | Ergotamine | 111.1 | 73.9 | 212.3 | **132.5** | 71.6 | 54 % | 80 % ± 39 % |
|  |  | Ergotaminine | 120.2 | 44.0 | 42.2 | **68.8** | 44.5 | 65 % | 47 % ± 20 % |

**Table S8** Results for the quantification of the twelve priority ergot alkaloids in rye flour 2 using the internal standard method

| **Sample** | **Laboratory - Method** | **Analyte** | **Mass fraction [µg/kg]** | | | **Avg. Mass fraction [µg/kg]** | **Std. Dev. [µg/kg]** | **Rel. Std. Dev.** | **Avg. Recovery Rate ± Std. Dev** |
| --- | --- | --- | --- | --- | --- | --- | --- | --- | --- |
|  |  |  | **1^st^ triplicate** | **2^nd^ triplicate** | **3^rd^ triplicate** |  |  |  |  |
| Rye flour 2 | BAM - IS | Ergocornine | 15.4 | 13.1 | 15.0 | **14.5** | 1.2 | 9 % | 87 % ± 4 % |
|  |  | Ergocorninine | 7.8 | 7.1 | 6.2 | **7.0** | 0.8 | 11 % | 79 % ± 4 % |
|  |  | Ergocristine | 25.9 | 26.4 | 27.6 | **26.7** | 0.9 | 3 % | 90 % ± 4 % |
|  |  | Ergocristinine | 8.1 | 8.7 | 9.0 | **8.6** | 0.5 | 6 % | 107 % ± 3 % |
|  |  | α/β-Ergocryptine | 18.7 | 18.4 | 17.6 | **18.2** | 0.5 | 3 % | 90 % ± 5 % |
|  |  | α/β-Ergocryptinine | 7.5 | 7.6 | 6.7 | **7.3** | 0.5 | 7 % | 92 % ± 9 % |
|  |  | Ergometrine | 7.8 | 7.9 | 8.4 | **8.0** | 0.3 | 4 % | 103 % ± 2 % |
|  |  | Ergometrinine | 1.2 | 1.4 | 1.4 | **1.3** | 0.1 | 7 % | 108 % ± 2 % |
|  |  | Ergosine | 19.6 | 19.9 | 22.8 | **20.8** | 1.8 | 8 % | 94 % ± 4 % |
|  |  | Ergosinine | 7.7 | 8.2 | 8.5 | **8.1** | 0.4 | 5 % | 83 % ± 3 % |
|  |  | Ergotamine | 36.2 | 36.0 | 38.6 | **36.9** | 1.5 | 4 % | 90 % ± 5 % |
|  |  | Ergotaminine | 9.9 | 10.1 | 11.2 | **10.4** | 0.7 | 7 % | 98 % ± 4 % |

**Table S9** Results for the quantification of the twelve priority ergot alkaloids in rye flour 2 using the standard addition method

| **Sample** | **Laboratory - Method** | **Analyte** | **Mass fraction [µg/kg]** | | | **Avg. Mass fraction [µg/kg]** | **Std. Dev. [µg/kg]** | **Rel. Std. Dev.** | **Avg. Recovery Rate ± Std. Dev** |
| --- | --- | --- | --- | --- | --- | --- | --- | --- | --- |
|  |  |  | **1^st^ triplicate** | **2^nd^ triplicate** | **3^rd^ triplicate** |  |  |  |  |
| Rye flour 2 | BAM - SA | Ergocornine | 17.3 | 10.8 | 17.6 | **15.3** | 3.9 | 25 % | 84 % ± 12 % |
|  |  | Ergocorninine | 8.6 | 6.9 | 6.3 | **7.3** | 1.2 | 17 % | 76 % ± 3 % |
|  |  | Ergocristine | 22.4 | 21.3 | 26.9 | **23.5** | 2.9 | 12 % | 101 % ± 6 % |
|  |  | Ergocristinine | 9.2 | 9.6 | 11.1 | **10.0** | 1.0 | 10 % | 90 % ± 3 % |
|  |  | α/β-Ergocryptine | 19.6 | 16.0 | 21.7 | **19.1** | 2.9 | 15 % | 86 % ± 10 % |
|  |  | α/β-Ergocryptinine | 8.2 | 6.3 | 7.1 | **7.2** | 0.9 | 13 % | 91 % ± 5 % |
|  |  | Ergometrine | 7.5 | 8.3 | 9.6 | **8.4** | 1.1 | 12 % | 97 % ± 9 % |
|  |  | Ergometrinine | 1.3 | 1.5 | 1.5 | **1.4** | 0.1 | 9 % | 103 % ± 3 % |
|  |  | Ergosine | 16.2 | 15.4 | 29.7 | **20.4** | 8.0 | 39 % | 102 % ± 25 % |
|  |  | Ergosinine | 7.7 | 8.4 | 10.3 | **8.8** | 1.4 | 15 % | 76 % ± 7 % |
|  |  | Ergotamine | 48.0 | 21.1 | 38.7 | **35.9** | 13.7 | 38 % | 101 % ± 37 % |
|  |  | Ergotaminine | 13.8 | 10.5 | 15.9 | **13.4** | 2.7 | 20 % | 76 % ± 11 % |

**Table S10** Results for the quantification of the twelve priority ergot alkaloids in wheat flour 1 using the internal standard method

| **Sample** | **Laboratory - Method** | **Analyte** | **Mass fraction [µg/kg]** | | | **Avg. Mass fraction [µg/kg]** | **Std. Dev. [µg/kg]** | **Rel. Std. Dev.** | **Avg. Recovery Rate ± Std. Dev** |
| --- | --- | --- | --- | --- | --- | --- | --- | --- | --- |
|  |  |  | **1^st^ triplicate** | **2^nd^ triplicate** | **3^rd^ triplicate** |  |  |  |  |
| Wheat flour 1 | BAM - IS | Ergocornine | 3.1 | 2.4 | 2.3 | **2.6** | 0.4 | 16 % | 96 % ± 3 % |
|  |  | Ergocorninine | 1.7 | 1.3 | 1.3 | **1.4** | 0.3 | 18 % | 91 % ± 2 % |
|  |  | Ergocristine | 7.1 | 6.5 | 5.9 | **6.5** | 0.6 | 9 % | 98 % ± 3 % |
|  |  | Ergocristinine | 2.7 | 2.5 | 2.5 | **2.5** | 0.1 | 4 % | 107 % ± 3 % |
|  |  | α/β-Ergocryptine | 3.6 | 3.1 | 3.0 | **3.2** | 0.3 | 10 % | 100 % ± 3 % |
|  |  | α/β-Ergocryptinine | 1.7 | 1.5 | 1.4 | **1.5** | 0.1 | 7 % | 98 % ± 4 % |
|  |  | Ergometrine | 2.1 | 1.8 | 1.9 | **2.0** | 0.2 | 9 % | 109 % ± 2 % |
|  |  | Ergometrinine | 0.5 | 0.4 | 0.5 | **0.5** | 0.0 | 7 % | 116 % ± 3 % |
|  |  | Ergosine | 8.0 | 7.5 | 7.8 | **7.8** | 0.2 | 3 % | 106 % ± 2 % |
|  |  | Ergosinine | 3.4 | 3.0 | 3.2 | **3.2** | 0.2 | 7 % | 80 % ± 2 % |
|  |  | Ergotamine | 10.0 | 10.7 | 9.9 | **10.2** | 0.5 | 4 % | 104 % ± 3 % |
|  |  | Ergotaminine | 3.0 | 3.0 | 3.0 | **3.0** | 0.0 | 0 % | 93 % ± 2 % |

**Table S11** Results for the quantification of the twelve priority ergot alkaloids in wheat flour 1 using the standard addition method

| **Sample** | **Laboratory - Method** | **Analyte** | **Mass fraction [µg/kg]** | | | **Avg. Mass fraction [µg/kg]** | **Std. Dev. [µg/kg]** | **Rel. Std. Dev.** | **Avg. Recovery Rate ± Std. Dev** |
| --- | --- | --- | --- | --- | --- | --- | --- | --- | --- |
|  |  |  | **1^st^ triplicate** | **2^nd^ triplicate** | **3^rd^ triplicate** |  |  |  |  |
| Wheat flour 1 | BAM - SA | Ergocornine | 3.5 | 2.7 | 2.4 | **2.9** | 0.6 | 20 % | 86 % ± 2 % |
|  |  | Ergocorninine | 1.8 | 1.3 | 1.2 | **1.4** | 0.3 | 21 % | 94 % ± 2 % |
|  |  | Ergocristine | 7.4 | 5.5 | 5.2 | **6.0** | 1.2 | 20 % | 106 % ± 10 % |
|  |  | Ergocristinine | 3.0 | 2.6 | 2.4 | **2.7** | 0.3 | 11 % | 102 % ± 6 % |
|  |  | α/β-Ergocryptine | 3.7 | 3.2 | 3.0 | **3.3** | 0.4 | 12 % | 97 % ± 1 % |
|  |  | α/β-Ergocryptinine | 1.8 | 1.5 | 1.4 | **1.6** | 0.2 | 14 % | 97 % ± 1 % |
|  |  | Ergometrine | 2.4 | 1.9 | 2.0 | **2.1** | 0.2 | 11 % | 102 % ± 2 % |
|  |  | Ergometrinine | 0.6 | 0.5 | 0.6 | **0.5** | 0.0 | 7 % | 108 % ± 1 % |
|  |  | Ergosine | 6.7 | 7.2 | 7.4 | **7.1** | 0.4 | 5 % | 116 % ± 11 % |
|  |  | Ergosinine | 3.0 | 2.7 | 2.8 | **2.8** | 0.2 | 7 % | 90 % ± 1 % |
|  |  | Ergotamine | 9.3 | 10.4 | 8.2 | **9.3** | 1.1 | 12 % | 114 % ± 6 % |
|  |  | Ergotaminine | 3.3 | 3.1 | 2.9 | **3.1** | 0.2 | 6 % | 90 % ± 4 % |

**Table S12** Results for the quantification of the twelve priority ergot alkaloids in wheat flour 2 using the internal standard method

| **Sample** | **Laboratory - Method** | **Analyte** | **Mass fraction [µg/kg]** | | | **Avg. Mass fraction [µg/kg]** | **Std. Dev. [µg/kg]** | **Rel. Std. Dev.** | **Avg. Recovery Rate ± Std. Dev** |
| --- | --- | --- | --- | --- | --- | --- | --- | --- | --- |
|  |  |  | **1^st^ triplicate** | **2^nd^ triplicate** | **3^rd^ triplicate** |  |  |  |  |
| Wheat flour 2 | BAM - IS | Ergocornine | 13.8 | 12.8 | 15.2 | **13.9** | 1.2 | 9 % | 94 % ± 2 % |
|  |  | Ergocorninine | 8.8 | 10.1 | 9.3 | **9.4** | 0.6 | 7 % | 90 % ± 3 % |
|  |  | Ergocristine | 25.0 | 24.3 | 26.2 | **25.2** | 0.9 | 4 % | 98 % ± 3 % |
|  |  | Ergocristinine | 11.1 | 11.2 | 11.5 | **11.3** | 0.2 | 2 % | 112 % ± 2 % |
|  |  | α/β-Ergocryptine | 16.9 | 15.7 | 16.9 | **16.5** | 0.7 | 4 % | 96 % ± 3 % |
|  |  | α/β-Ergocryptinine | 8.9 | 8.7 | 9.0 | **8.9** | 0.1 | 1 % | 101 % ± 4 % |
|  |  | Ergometrine | 8.9 | 8.7 | 9.2 | **8.9** | 0.2 | 3 % | 101 % ± 2 % |
|  |  | Ergometrinine | 2.2 | 2.1 | 2.2 | **2.2** | 0.1 | 3 % | 104 % ± 1 % |
|  |  | Ergosine | 34.0 | 30.7 | 33.9 | **32.9** | 1.9 | 6 % | 97 % ± 2 % |
|  |  | Ergosinine | 14.4 | 14.0 | 15.0 | **14.5** | 0.5 | 3 % | 83 % ± 2 % |
|  |  | Ergotamine | 37.7 | 38.0 | 35.3 | **37.0** | 1.5 | 4 % | 98 % ± 3 % |
|  |  | Ergotaminine | 12.5 | 12.8 | 12.5 | **12.6** | 0.2 | 2 % | 99 % ± 2 % |

**Table S13** Results for the quantification of the twelve priority ergot alkaloids in wheat flour 2 using the standard addition method

| **Sample** | **Laboratory - Method** | **Analyte** | **Mass fraction [µg/kg]** | | | **Avg. Mass fraction [µg/kg]** | **Std. Dev. [µg/kg]** | **Rel. Std. Dev.** | **Avg. Recovery Rate ± Std. Dev** |
| --- | --- | --- | --- | --- | --- | --- | --- | --- | --- |
|  |  |  | **1^st^ triplicate** | **2^nd^ triplicate** | **3^rd^ triplicate** |  |  |  |  |
| Wheat flour 2 | BAM - SA | Ergocornine | 11.2 | 10.4 | 18.3 | **13.3** | 4.3 | 33 % | 102 % ± 21 % |
|  |  | Ergocorninine | 7.3 | 8.8 | 8.0 | **8.0** | 0.7 | 9 % | 104 % ± 6 % |
|  |  | Ergocristine | 18.6 | 19.6 | 26.2 | **21.5** | 4.1 | 19 % | 116 % ± 19 % |
|  |  | Ergocristinine | 10.9 | 10.6 | 12.5 | **11.3** | 1.0 | 9 % | 109 % ± 6 % |
|  |  | α/β-Ergocryptine | 15.7 | 12.3 | 15.9 | **14.6** | 2.0 | 14 % | 108 % ± 9 % |
|  |  | α/β-Ergocryptinine | 8.8 | 8.0 | 9.7 | **8.8** | 0.8 | 10 % | 100 % ± 5 % |
|  |  | Ergometrine | 8.9 | 8.2 | 9.3 | **8.8** | 0.6 | 6 % | 100 % ± 4 % |
|  |  | Ergometrinine | 2.2 | 2.1 | 2.3 | **2.2** | 0.1 | 6 % | 103 % ± 2 % |
|  |  | Ergosine | 25.7 | 18.9 | 33.4 | **26.0** | 7.3 | 28 % | 127 % ± 29 % |
|  |  | Ergosinine | 14.5 | 12.1 | 15.0 | **13.8** | 1.5 | 11 % | 86 % ± 6 % |
|  |  | Ergotamine | 29.8 | 28.2 | 28.6 | **28.9** | 0.8 | 3 % | 124 % ± 2 % |
|  |  | Ergotaminine | 16.1 | 15.5 | 14.0 | **15.2** | 1.1 | 7 % | 81 % ± 5 % |

**Table S14** Results for the quantification of the twelve priority ergot alkaloids in wholemeal bread using the internal standard method

| **Sample** | **Laboratory - Method** | **Analyte** | **Mass fraction [µg/kg]** | | | **Avg. Mass fraction [µg/kg]** | **Std. Dev. [µg/kg]** | **Rel. Std. Dev.** | **Avg. Recovery Rate ± Std. Dev** |
| --- | --- | --- | --- | --- | --- | --- | --- | --- | --- |
|  |  |  | **1^st^ triplicate** | **2^nd^ triplicate** | **3^rd^ triplicate** |  |  |  |  |
| Wholemeal bread | BAM - IS | Ergocornine | 0.3 | 0.3 | 0.3 | **0.3** | 0.0 | 3 % | 85 % ± 3 % |
|  |  | Ergocorninine | 0.2 | 0.3 | 0.3 | **0.3** | 0.0 | 16 % | 86 % ± 2 % |
|  |  | Ergocristine | 9.4 | 7.9 | 9.9 | **9.1** | 1.0 | 11 % | 95 % ± 2 % |
|  |  | Ergocristinine | 5.3 | 4.5 | 5.7 | **5.2** | 0.6 | 11 % | 96 % ± 2 % |
|  |  | α/β-Ergocryptine | 0.8 | 0.7 | 0.9 | **0.8** | 0.1 | 9 % | 88 % ± 2 % |
|  |  | α/β-Ergocryptinine | 0.6 | 0.5 | 0.6 | **0.6** | 0.1 | 10 % | 87 % ± 3 % |
|  |  | Ergometrine | 1.4 | 1.4 | 1.5 | **1.4** | 0.1 | 4 % | 75 % ± 2 % |
|  |  | Ergometrinine | 2.0 | 2.1 | 2.2 | **2.1** | 0.1 | 5 % | 91 % ± 2 % |
|  |  | Ergosine | 8.6 | 11.3 | 8.7 | **9.5** | 1.5 | 16 % | 85 % ± 2 % |
|  |  | Ergosinine | 5.1 | 6.8 | 5.1 | **5.7** | 1.0 | 17 % | 91 % ± 1 % |
|  |  | Ergotamine | 6.0 | 5.6 | 6.2 | **5.9** | 0.3 | 5 % | 82 % ± 2 % |
|  |  | Ergotaminine | 2.9 | 2.8 | 3.0 | **2.9** | 0.1 | 4 % | 91 % ± 2 % |

**Table S15** Results for the quantification of the twelve priority ergot alkaloids in wholemeal bread using the standard addition method

| **Sample** | **Laboratory - Method** | **Analyte** | **Mass fraction [µg/kg]** | | | **Avg. Mass fraction [µg/kg]** | **Std. Dev. [µg/kg]** | **Rel. Std. Dev.** | **Avg. Recovery Rate ± Std. Dev** |
| --- | --- | --- | --- | --- | --- | --- | --- | --- | --- |
|  |  |  | **1^st^ triplicate** | **2^nd^ triplicate** | **3^rd^ triplicate** |  |  |  |  |
| Wholemeal bread | BAM - SA | Ergocornine | 0.4 | 0.4 | 0.3 | **0.4** | 0.0 | 9 % | 86 % ± 8 % |
|  |  | Ergocorninine | 0.3 | 0.2 | 0.2 | **0.2** | 0.0 | 9 % | 96 % ± 5 % |
|  |  | Ergocristine | 4.0 | 1.3 | 8.0 | **4.4** | 3.4 | 76 % | 305 % ± 240 % |
|  |  | Ergocristinine | 4.8 | 4.7 | 6.8 | **5.4** | 1.2 | 22 % | 91 % ± 13 % |
|  |  | α/β-Ergocryptine | 0.8 | 0.7 | 0.8 | **0.8** | 0.1 | 10 % | 93 % ± 1 % |
|  |  | α/β-Ergocryptinine | 0.6 | 0.5 | 0.6 | **0.6** | 0.1 | 11 % | 95 % ± 3 % |
|  |  | Ergometrine | 1.4 | 1.5 | 1.6 | **1.5** | 0.1 | 7 % | 75 % ± 2 % |
|  |  | Ergometrinine | 2.0 | 2.2 | 2.3 | **2.2** | 0.2 | 8 % | 91 % ± 3 % |
|  |  | Ergosine | 6.1 | 14.8 | 9.6 | **10.2** | 4.4 | 43 % | 87 % ± 29 % |
|  |  | Ergosinine | 4.3 | 8.4 | 5.4 | **6.0** | 2.1 | 35 % | 88 % ± 18 % |
|  |  | Ergotamine | 4.3 | 3.6 | 6.3 | **4.7** | 1.4 | 30 % | 107 % ± 22 % |
|  |  | Ergotaminine | 2.5 | 2.2 | 3.0 | **2.5** | 0.4 | 17 % | 105 % ± 11 |

**Table S16** Results for the quantification of the twelve priority ergot alkaloids in mixed bread using the internal standard method

| **Sample** | **Laboratory - Method** | **Analyte** | **Mass fraction [µg/kg]** | | | **Avg. Mass fraction [µg/kg]** | **Std. Dev. [µg/kg]** | **Rel. Std. Dev.** | **Avg. Recovery Rate ± Std. Dev** |
| --- | --- | --- | --- | --- | --- | --- | --- | --- | --- |
|  |  |  | **1^st^ triplicate** | **2^nd^ triplicate** | **3^rd^ triplicate** |  |  |  |  |
| Mixed bread | BAM - IS | Ergocornine | 2.0 | 2.1 | 2.1 | **2.1** | 0.0 | 2 % | 84 % ± 1 % |
|  |  | Ergocorninine | 2.0 | 2.0 | 2.2 | **2.1** | 0.1 | 4 % | 83 % ± 2 % |
|  |  | Ergocristine | 4.3 | 4.3 | 4.6 | **4.4** | 0.2 | 4 % | 88 % ± 1 % |
|  |  | Ergocristinine | 2.9 | 2.9 | 3.1 | **3.0** | 0.2 | 5 % | 95 % ± 1 % |
|  |  | α/β-Ergocryptine | 3.8 | 3.8 | 3.9 | **3.8** | 0.1 | 1 % | 87 % ± 1 % |
|  |  | α/β-Ergocryptinine | 3.0 | 3.1 | 3.2 | **3.1** | 0.1 | 2 % | 85 % ± 1 % |
|  |  | Ergometrine | 1.2 | 1.2 | 1.2 | **1.2** | 0.0 | 1 % | 81 % ± 0 % |
|  |  | Ergometrinine | 1.2 | 1.2 | 1.2 | **1.2** | 0.0 | 3 % | 96 % ± 1 % |
|  |  | Ergosine | 3.5 | 3.7 | 3.7 | **3.6** | 0.1 | 4 % | 83 % ± 1 % |
|  |  | Ergosinine | 2.1 | 2.3 | 2.2 | **2.2** | 0.1 | 3 % | 91 % ± 0 % |
|  |  | Ergotamine | 7.3 | 6.6 | 6.8 | **6.9** | 0.4 | 5 % | 85 % ± 0 % |
|  |  | Ergotaminine | 3.9 | 3.6 | 3.6 | **3.7** | 0.2 | 5 % | 90 % ± 0 % |

**Table S17** Results for the quantification of the twelve priority ergot alkaloids in mixed bread using the standard addition method

| **Sample** | **Laboratory - Method** | **Analyte** | **Mass fraction [µg/kg]** | | | **Avg. Mass fraction [µg/kg]** | **Std. Dev. [µg/kg]** | **Rel. Std. Dev.** | **Avg. Recovery Rate ± Std. Dev** |
| --- | --- | --- | --- | --- | --- | --- | --- | --- | --- |
|  |  |  | **1^st^ triplicate** | **2^nd^ triplicate** | **3^rd^ triplicate** |  |  |  |  |
| Mixed bread | BAM - SA | Ergocornine | 2.2 | 2.4 | 2.2 | **2.2** | 0.1 | 5 % | 78 % ± 3 % |
|  |  | Ergocorninine | 1.9 | 1.9 | 2.0 | **1.9** | 0.1 | 3 % | 81 % ± 1 % |
|  |  | Ergocristine | 4.4 | 4.3 | 4.9 | **4.5** | 0.3 | 6 % | 85 % ± 1 % |
|  |  | Ergocristinine | 3.1 | 3.1 | 3.4 | **3.2** | 0.2 | 6 % | 87 % ± 1 % |
|  |  | α/β-Ergocryptine | 4.1 | 4.1 | 4.1 | **4.1** | 0.0 | 1 % | 80 % ± 1 % |
|  |  | α/β-Ergocryptinine | 3.1 | 3.2 | 3.1 | **3.1** | 0.0 | 2 % | 84 % ± 2 % |
|  |  | Ergometrine | 1.3 | 1.3 | 1.3 | **1.3** | 0.0 | 1 % | 77 % ± 1 % |
|  |  | Ergometrinine | 1.3 | 1.3 | 1.3 | **1.3** | 0.0 | 4 % | 91 % ± 2 % |
|  |  | Ergosine | 3.6 | 3.9 | 3.7 | **3.7** | 0.2 | 4 % | 81 % ± 2 % |
|  |  | Ergosinine | 2.2 | 2.4 | 2.3 | **2.3** | 0.1 | 4 % | 88 % ± 2 % |
|  |  | Ergotamine | 8.4 | 6.9 | 7.1 | **7.5** | 0.8 | 11 % | 78 % ± 4 % |
|  |  | Ergotaminine | 4.3 | 3.8 | 3.8 | **4.0** | 0.3 | 7 % | 84 % ± 2 % |

# **Quantification results Eurofins**

**Table S18** Results for the quantification of the twelve priority ergot alkaloids in rye flour 1 using the internal standard method

| **Sample** | **Laboratory - Method** | **Analyte** | **Mass fraction [µg/kg]** | | | **Avg. Mass fraction [µg/kg]** | **Std. Dev. [µg/kg]** | **Rel. Std. Dev.** | **Avg. Recovery Rate ± Std. Dev** |
| --- | --- | --- | --- | --- | --- | --- | --- | --- | --- |
|  |  |  | **1^st^ triplicate** | **2^nd^ triplicate** | **3^rd^ triplicate** |  |  |  |  |
| Rye flour 1 | Eurofins - IS | Ergocornine | 17.2 | 19.4 | 18.1 | **18.3** | 1.1 | 6% | 66 % ± 7 % |
|  |  | Ergocorninine | 5.5 | 5.9 | 6.5 | **5.9** | 0.5 | 9% | 74 % ± 22 % |
|  |  | Ergocristine | 81.8 | 74.1 | 74.1 | **76.7** | 4.4 | 6% | 77 % ± 14 % |
|  |  | Ergocristinine | 17.4 | 14.3 | 17.1 | **16.3** | 1.7 | 10% | 95 % ± 5 % |
|  |  | α/β-Ergocryptine | 31.9 | 31.8 | 36.6 | **33.4** | 2.8 | 8% | 62 % ± 5 % |
|  |  | α/β-Ergocryptinine | 8.3 | 8.1 | 10.5 | **9.0** | 1.3 | 15% | 87 % ± 4 % |
|  |  | Ergometrine | 13.5 | 11.5 | 13.6 | **12.9** | 1.2 | 9% | 101 % ± 6 % |
|  |  | Ergometrinine | 1.7 | 1.5 | 1.7 | **1.6** | 0.1 | 8% | 134 % ± 6 % |
|  |  | Ergosine | 80.6 | 89.8 | 79.4 | **83.3** | 5.7 | 7% | 90 % ± 15 % |
|  |  | Ergosinine | 21.1 | 24.4 | 20.4 | **22.0** | 2.1 | 10% | 85 % ± 10 % |
|  |  | Ergotamine | 102.2 | 84.9 | 95.7 | **94.3** | 8.7 | 9% | 86 % ± 8 % |
|  |  | Ergotaminine | 18.7 | 18.8 | 19.0 | **18.8** | 0.2 | 1% | 93 % ± 16 % |

**Table S19** Results for the quantification of the twelve priority ergot alkaloids in rye flour 1 using the standard addition method

| **Sample** | **Laboratory - Method** | **Analyte** | **Mass fraction [µg/kg]** | | | **Avg. Mass fraction [µg/kg]** | **Std. Dev. [µg/kg]** | **Rel. Std. Dev.** | **Avg. Recovery Rate ± Std. Dev** |
| --- | --- | --- | --- | --- | --- | --- | --- | --- | --- |
|  |  |  | **1^st^ triplicate** | **2^nd^ triplicate** | **3^rd^ triplicate** |  |  |  |  |
| Rye flour 1 | Eurofins - SA | Ergocornine | 11.9 | 13.1 | 11.5 | **12.1** | 0.8 | 7% | 99 % ± 8 % |
|  |  | Ergocorninine | 4.8 | 2.9 | 4.1 | **3.9** | 1.0 | 24% | 111 % ± 7 % |
|  |  | Ergocristine | 36.3 | 13.4 | 20.7 | **23.5** | 11.7 | 50% | 275 % ± 80 % |
|  |  | Ergocristinine | 17.4 | 14.3 | 17.1 | **16.3** | 1.7 | 10% | 125 % ± 32 % |
|  |  | α/β-Ergocryptine | 17.8 | 14.0 | 29.9 | **20.5** | 8.3 | 40% | 109 % ± 30 % |
|  |  | α/β-Ergocryptinine | 7.2 | 6.8 | 11.1 | **8.4** | 2.4 | 29% | 95 % ± 14 % |
|  |  | Ergometrine | 19.4 | 11.1 | 17.5 | **16.0** | 4.3 | 27% | 84 % ± 15 % |
|  |  | Ergometrinine | 2.2 | 2.0 | 2.2 | **2.1** | 0.1 | 7% | 102 % ± 6 % |
|  |  | Ergosine | 220.6 | 34.0 | 23.5 | **92.7** | 110.9 | 120% | 177 % ± 121 % |
|  |  | Ergosinine | 19.2 | 17.0 | 13.6 | **16.6** | 2.9 | 17% | 113 % ± 6 % |
|  |  | Ergotamine | 80.3 | 22.2 | 46.4 | **49.6** | 29.2 | 59% | 201 % ± 101 % |
|  |  | Ergotaminine | 19.4 | 11.4 | 18.2 | **16.3** | 4.3 | 26% | 111 % ± 24 % |

**Table S20** Results for the quantification of the twelve priority ergot alkaloids in rye flour 2 using the internal standard method

| **Sample** | **Laboratory - Method** | **Analyte** | **Mass fraction [µg/kg]** | | | **Avg. Mass fraction [µg/kg]** | **Std. Dev. [µg/kg]** | **Rel. Std. Dev.** | **Avg. Recovery Rate ± Std. Dev** |
| --- | --- | --- | --- | --- | --- | --- | --- | --- | --- |
|  |  |  | **1^st^ triplicate** | **2^nd^ triplicate** | **3^rd^ triplicate** |  |  |  |  |
| Rye flour 2 | Eurofins - IS | Ergocornine | 15.4 | 15.7 | 15.4 | **15.5** | 0.2 | 1% | 65 % ± 5 % |
|  |  | Ergocorninine | 5.8 | 6.2 | 5.8 | **5.9** | 0.2 | 4% | 78 % ± 14 % |
|  |  | Ergocristine | 26.1 | 24.1 | 27.5 | **25.9** | 1.7 | 7% | 83 % ± 3 % |
|  |  | Ergocristinine | 7.7 | 6.9 | 7.3 | **7.3** | 0.4 | 5% | 109 % ± 6 % |
|  |  | α/β-Ergocryptine | 20.2 | 19.8 | 21.3 | **20.4** | 0.8 | 4% | 61 % ± 2 % |
|  |  | α/β-Ergocryptinine | 8.2 | 7.1 | 7.1 | **7.5** | 0.6 | 8% | 96 % ± 17 % |
|  |  | Ergometrine | 8.7 | 8.7 | 8.6 | **8.7** | 0.1 | 1% | 103 % ± 6 % |
|  |  | Ergometrinine | 1.3 | 1.3 | 1.2 | **1.2** | 0.0 | 3% | 132 % ± 3 % |
|  |  | Ergosine | 22.4 | 18.1 | 20.9 | **20.5** | 2.2 | 11% | 92 % ± 9 % |
|  |  | Ergosinine | 7.0 | 4.9 | 6.3 | **6.1** | 1.1 | 18% | 89 % ± 5 % |
|  |  | Ergotamine | 34.3 | 32.6 | 35.3 | **34.1** | 1.3 | 4% | 94 % ± 7 % |
|  |  | Ergotaminine | 8.6 | 7.1 | 7.5 | **7.7** | 0.7 | 10% | 84 % ± 27 % |

**Table S21** Results for the quantification of the twelve priority ergot alkaloids in rye flour 2 using the standard addition method

| **Sample** | **Laboratory - Method** | **Analyte** | **Mass fraction [µg/kg]** | | | **Avg. Mass fraction [µg/kg]** | **Std. Dev. [µg/kg]** | **Rel. Std. Dev.** | **Avg. Recovery Rate ± Std. Dev** |
| --- | --- | --- | --- | --- | --- | --- | --- | --- | --- |
|  |  |  | **1^st^ triplicate** | **2^nd^ triplicate** | **3^rd^ triplicate** |  |  |  |  |
| Rye flour 2 | Eurofins - SA | Ergocornine | 11.9 | 12.2 | 24.0 | **16.0** | 6.9 | 43% | 68 % ± 20 % |
|  |  | Ergocorninine | 5.2 | 4.1 | 8.2 | **5.8** | 2.2 | 37% | 83 % ± 17 % |
|  |  | Ergocristine | 24.4 | 23.1 | 23.9 | **23.8** | 0.7 | 3% | 86 % ± 1 % |
|  |  | Ergocristinine | 7.7 | 6.9 | 7.3 | **7.3** | 0.4 | 5% | 91 % ± 11 % |
|  |  | α/β-Ergocryptine | 15.3 | 16.5 | 22.7 | **18.2** | 4.0 | 22% | 69 % ± 10 % |
|  |  | α/β-Ergocryptinine | 6.6 | 6.5 | 9.4 | **7.5** | 1.7 | 22% | 96 % ± 9 % |
|  |  | Ergometrine | 11.2 | 10.5 | 13.1 | **11.6** | 1.4 | 12% | 77 % ± 7 % |
|  |  | Ergometrinine | 1.5 | 1.8 | 1.5 | **1.6** | 0.2 | 9% | 101 % ± 9 % |
|  |  | Ergosine | 40.2 | 10.4 | 68.8 | **39.8** | 29.2 | 73% | 78 % ± 67 % |
|  |  | Ergosinine | 8.6 | 5.2 | 8.5 | **7.5** | 1.9 | 26% | 74 % ± 12 % |
|  |  | Ergotamine | 19.8 | 13.5 | 35.9 | **23.1** | 11.5 | 50% | 185 % ± 41 % |
|  |  | Ergotaminine | 8.5 | 3.1 | 20.5 | **10.7** | 8.9 | 83% | 85 % ± 48 % |

**Table S22** Results for the quantification of the twelve priority ergot alkaloids in wheat flour 1 using the internal standard method

| **Sample** | **Laboratory - Method** | **Analyte** | **Mass fraction [µg/kg]** | | | **Avg. Mass fraction [µg/kg]** | **Std. Dev. [µg/kg]** | **Rel. Std. Dev.** | **Avg. Recovery Rate ± Std. Dev** |
| --- | --- | --- | --- | --- | --- | --- | --- | --- | --- |
|  |  |  | **1^st^ triplicate** | **2^nd^ triplicate** | **3^rd^ triplicate** |  |  |  |  |
| Wheat flour 1 | Eurofins - IS | Ergocornine | 2.4 | 2.3 | 3.4 | **2.7** | 0.6 | 23% | 58 % ± 2 % |
|  |  | Ergocorninine | 1.2 | 1.2 | 1.5 | **1.3** | 0.2 | 13% | 76 % ± 9 % |
|  |  | Ergocristine | 7.0 | 6.7 | 5.9 | **6.6** | 0.6 | 8% | 78 % ± 3 % |
|  |  | Ergocristinine | 1.8 | 1.8 | 1.7 | **1.7** | 0.0 | 2% | 106 % ± 8 % |
|  |  | α/β-Ergocryptine | 3.7 | 4.2 | 5.0 | **4.3** | 0.6 | 15% | 44 % ± 2 % |
|  |  | α/β-Ergocryptinine | 1.5 | 1.6 | 2.0 | **1.7** | 0.3 | 15% | 92 % ± 8 % |
|  |  | Ergometrine | 1.7 | 2.0 | 2.2 | **1.9** | 0.2 | 13% | 106 % ± 3 % |
|  |  | Ergometrinine | 0.4 | 0.4 | 0.4 | **0.4** | 0.0 | 2% | 124 % ± 2 % |
|  |  | Ergosine | 5.4 | 6.2 | 6.6 | **6.1** | 0.6 | 10% | 89 % ± 5 % |
|  |  | Ergosinine | 1.8 | 1.6 | 2.1 | **1.8** | 0.3 | 14% | 81 % ± 3 % |
|  |  | Ergotamine | 9.5 | 9.2 | 9.9 | **9.5** | 0.4 | 4% | 84 % ± 6 % |
|  |  | Ergotaminine | 1.8 | 2.4 | 2.8 | **2.4** | 0.5 | 21% | 76 % ± 13 % |

**Table S23** Results for the quantification of the twelve priority ergot alkaloids in wheat flour 1 using the standard addition method

| **Sample** | **Laboratory - Method** | **Analyte** | **Mass fraction [µg/kg]** | | | **Avg. Mass fraction [µg/kg]** | **Std. Dev. [µg/kg]** | **Rel. Std. Dev.** | **Avg. Recovery Rate ± Std. Dev** |
| --- | --- | --- | --- | --- | --- | --- | --- | --- | --- |
|  |  |  | **1^st^ triplicate** | **2^nd^ triplicate** | **3^rd^ triplicate** |  |  |  |  |
| Wheat flour 1 | Eurofins - SA | Ergocornine | 2.4 | 2.2 | 3.7 | **2.7** | 0.8 | 29% | 57 % ± 4 % |
|  |  | Ergocorninine | 1.2 | 1.0 | 1.5 | **1.2** | 0.2 | 18% | 80 % ± 5 % |
|  |  | Ergocristine | 8.2 | 7.0 | 6.1 | **7.1** | 1.1 | 15% | 73 % ± 7 % |
|  |  | Ergocristinine | 1.8 | 1.8 | 1.7 | **1.7** | 0.0 | 2% | 87 % ± 4 % |
|  |  | α/β-Ergocryptine | 3.6 | 3.6 | 4.6 | **3.9** | 0.6 | 14% | 48 % ± 3 % |
|  |  | α/β-Ergocryptinine | 1.8 | 1.7 | 2.2 | **1.9** | 0.3 | 13% | 83 % ± 2 % |
|  |  | Ergometrine | 2.1 | 2.2 | 2.7 | **2.3** | 0.3 | 14% | 89 % ± 3 % |
|  |  | Ergometrinine | 0.5 | 0.5 | 0.5 | **0.5** | 0.0 | 1% | 98 % ± 1 % |
|  |  | Ergosine | 5.2 | 6.9 | 7.9 | **6.7** | 1.4 | 21% | 82 % ± 14 % |
|  |  | Ergosinine | 2.0 | 1.5 | 2.4 | **2.0** | 0.4 | 22% | 75 % ± 5 % |
|  |  | Ergotamine | 9.4 | 9.7 | 14.4 | **11.1** | 2.8 | 25% | 74 % ± 12 % |
|  |  | Ergotaminine | 1.9 | 2.0 | 2.3 | **2.1** | 0.2 | 8% | 85 % ± 9 % |

**Table S24** Results for the quantification of the twelve priority ergot alkaloids in wheat flour 2 using the internal standard method

| **Sample** | **Laboratory - Method** | **Analyte** | **Mass fraction [µg/kg]** | | | **Avg. Mass fraction [µg/kg]** | **Std. Dev. [µg/kg]** | **Rel. Std. Dev.** | **Avg. Recovery Rate ± Std. Dev** |
| --- | --- | --- | --- | --- | --- | --- | --- | --- | --- |
|  |  |  | **1^st^ triplicate** | **2^nd^ triplicate** | **3^rd^ triplicate** |  |  |  |  |
| Wheat flour 2 | Eurofins - IS | Ergocornine | 14.5 | 17.0 | 14.7 | **15.4** | 1.4 | 9% | 53 % ± 5 % |
|  |  | Ergocorninine | 8.6 | 9.7 | 8.7 | **9.0** | 0.6 | 7% | 91 % ± 22 % |
|  |  | Ergocristine | 25.3 | 26.1 | 25.0 | **25.5** | 0.6 | 2% | 81 % ± 6 % |
|  |  | Ergocristinine | 9.3 | 9.3 | 9.2 | **9.3** | 0.1 | 1% | 104 % ± 5 % |
|  |  | α/β-Ergocryptine | 20.8 | 20.6 | 22.6 | **21.3** | 1.1 | 5% | 43 % ± 1 % |
|  |  | α/β-Ergocryptinine | 8.2 | 8.2 | 8.8 | **8.4** | 0.4 | 4% | 96 % ± 8 % |
|  |  | Ergometrine | 8.6 | 9.6 | 9.7 | **9.3** | 0.6 | 6% | 103 % ± 8 % |
|  |  | Ergometrinine | 1.9 | 2.2 | 2.2 | **2.1** | 0.2 | 9% | 120 % ± 2 % |
|  |  | Ergosine | 33.5 | 33.4 | 30.5 | **32.5** | 1.7 | 5% | 91 % ± 6 % |
|  |  | Ergosinine | 10.0 | 10.3 | 10.0 | **10.1** | 0.2 | 2% | 85 % ± 6 % |
|  |  | Ergotamine | 33.6 | 37.2 | 36.9 | **35.9** | 2.0 | 6% | 89 % ± 8 % |
|  |  | Ergotaminine | 9.7 | 9.3 | 9.8 | **9.6** | 0.3 | 3% | 91 % ± 36 % |

**Table S25** Results for the quantification of the twelve priority ergot alkaloids in wheat flour 2 using the standard addition method

| **Sample** | **Laboratory - Method** | **Analyte** | **Mass fraction [µg/kg]** | | | **Avg. Mass fraction [µg/kg]** | **Std. Dev. [µg/kg]** | **Rel. Std. Dev.** | **Avg. Recovery Rate ± Std. Dev** |
| --- | --- | --- | --- | --- | --- | --- | --- | --- | --- |
|  |  |  | **1^st^ triplicate*** | **2^nd^ triplicate** | **3^rd^ triplicate** |  |  |  |  |
| Wheat flour 2 | Eurofins - SA | Ergocornine | 21.3 | 24.6 | 15.4 | **20.0** | 6.5 | 32% | 41 % ± 8 % |
|  |  | Ergocorninine | 26.9 | 11.2 | 13.2 | **12.2** | 1.4 | 11% | 53 % ± 14 % |
|  |  | Ergocristine | 60.6 | 30.8 | 33.9 | **32.4** | 2.2 | 7% | 53 % ± 15 % |
|  |  | Ergocristinine | 9.3 | 9.3 | 9.2 | **9.2** | 0.1 | 1% | 81 % ± 6 % |
|  |  | α/β-Ergocryptine | 19.5 | 23.8 | 27.6 | **25.7** | 2.7 | 10% | 39 % ± 6 % |
|  |  | α/β-Ergocryptinine | 11.5 | 9.2 | 10.5 | **9.8** | 1.0 | 10% | 78 % ± 3 % |
|  |  | Ergometrine | 21.3 | 19.3 | 11.8 | **15.5** | 5.4 | 34% | 59 % ± 19 % |
|  |  | Ergometrinine | 2.7 | 3.4 | 2.8 | **3.1** | 0.4 | 14% | 86 % ± 9 % |
|  |  | Ergosine | 2263.0 | 54.4 | 38.9 | **46.7** | 10.9 | 23% | 42 % ± 37 % |
|  |  | Ergosinine | 15.6 | 13.8 | 10.8 | **12.3** | 2.1 | 17% | 65 % ± 9 % |
|  |  | Ergotamine | 81.1 | 48.6 | 53.4 | **51.0** | 3.4 | 7% | 55 % ± 13 % |
|  |  | Ergotaminine | 82.3 | 6.7 | 14.8 | **10.7** | 5.7 | 53% | 50 % ± 32 % |

** Outlier was not used for the calculation of Std. Dev., Rel. Std. Dev, and Avg. Recovery Rate.*

**Table S26** Results for the quantification of the twelve priority ergot alkaloids in wholemeal bread using the internal standard method

| **Sample** | **Laboratory - Method** | **Analyte** | **Mass fraction [µg/kg]** | | | **Avg. Mass fraction [µg/kg]** | **Std. Dev. [µg/kg]** | **Rel. Std. Dev.** | **Avg. Recovery Rate ± Std. Dev** |
| --- | --- | --- | --- | --- | --- | --- | --- | --- | --- |
|  |  |  | **1^st^ triplicate** | **2^nd^ triplicate** | **3^rd^ triplicate** |  |  |  |  |
| Wholemeal bread | Eurofins - IS | Ergocornine | *n.d.* | *n.d.* | *n.d.* | **-** | - | - | - |
|  |  | Ergocorninine | 0.3 | 0.7 | 0.7 | **0.6** | 0.2 | 37% | 80 % ± 20 % |
|  |  | Ergocristine | 15.4 | 15.9 | 6.6 | **12.6** | 5.2 | 41% | 87 % ± 6 % |
|  |  | Ergocristinine | 7.6 | 8.3 | 4.4 | **6.8** | 2.1 | 31% | 102 % ± 10 % |
|  |  | α/β-Ergocryptine | 1.2 | 1.1 | 1.1 | **1.2** | 0.1 | 5% | 87 % ± 7 % |
|  |  | α/β-Ergocryptinine | 0.5 | 0.4 | 0.4 | **0.4** | 0.1 | 12% | 89 % ± 11 % |
|  |  | Ergometrine | 1.0 | 1.0 | 0.9 | **0.9** | 0.0 | 3% | 90 % ± 5 % |
|  |  | Ergometrinine | 1.5 | 1.5 | 1.4 | **1.5** | 0.1 | 6% | 121 % ± 1 % |
|  |  | Ergosine | 5.5 | 5.9 | 6.2 | **5.9** | 0.4 | 6% | 84 % ± 8 % |
|  |  | Ergosinine | 3.7 | 4.1 | 4.8 | **4.2** | 0.5 | 13% | 93 % ± 8 % |
|  |  | Ergotamine | 5.2 | 5.6 | 3.8 | **4.8** | 0.9 | 19% | 93 % ± 13 % |
|  |  | Ergotaminine | 2.8 | 2.5 | 1.8 | **2.4** | 0.5 | 21% | 83 % ± 21 % |
| *n.d. – not detected* | | | | | | | | | |

**Table S27** Results for the quantification of the twelve priority ergot alkaloids in wholemeal bread using the standard addition method

| **Sample** | **Laboratory - Method** | **Analyte** | **Mass fraction [µg/kg]** | | | **Avg. Mass fraction [µg/kg]** | **Std. Dev. [µg/kg]** | **Rel. Std. Dev.** | **Avg. Recovery Rate ± Std. Dev** |
| --- | --- | --- | --- | --- | --- | --- | --- | --- | --- |
|  |  |  | **1^st^ triplicate** | **2^nd^ triplicate** | **3^rd^ triplicate** |  |  |  |  |
| Wholemeal bread | Eurofins - SA | Ergocornine | *n.d.* | *n.d.* | *n.d.* | **-** | - | - | - |
|  |  | Ergocorninine | 0.4 | 1.2 | 0.6 | **0.7** | 0.4 | 58% | 68 % ± 6 % |
|  |  | Ergocristine | 70.7 | 31.5 | 7.3 | **36.5** | 32.0 | 88% | 46 % ± 26 % |
|  |  | Ergocristinine | 7.6 | 8.3 | 4.4 | **6.8** | 2.1 | 31% | 59 % ± 15 % |
|  |  | α/β-Ergocryptine | 1.5 | 1.5 | 1.2 | **1.4** | 0.2 | 12% | 69 % ± 3 % |
|  |  | α/β-Ergocryptinine | 0.5 | 0.5 | 0.4 | **0.5** | 0.1 | 21% | 79 % ± 2 % |
|  |  | Ergometrine | 1.1 | 1.1 | 0.9 | **1.0** | 0.1 | 11% | 83 % ± 3 % |
|  |  | Ergometrinine | 1.6 | 1.6 | 1.5 | **1.6** | 0.1 | 6% | 112 % ± 2 % |
|  |  | Ergosine | 9.0 | 8.3 | 4.2 | **7.2** | 2.6 | 36% | 76 % ± 32 % |
|  |  | Ergosinine | 4.9 | 5.3 | 3.3 | **4.5** | 1.1 | 23% | 91 % ± 27 % |
|  |  | Ergotamine | 10.8 | 9.6 | 3.8 | **8.0** | 3.7 | 46% | 62 % ± 15 % |
|  |  | Ergotaminine | 3.2 | 4.5 | 1.9 | **3.2** | 1.3 | 40% | 63 % ± 3 % |
| *n.d. – not detected* | | | | | | | | | |

**Table S28** Results for the quantification of the twelve priority ergot alkaloids in mixed bread using the internal addition method

| **Sample** | **Laboratory - Method** | **Analyte** | **Mass fraction [µg/kg]** | | | **Avg. Mass fraction [µg/kg]** | **Std. Dev. [µg/kg]** | **Rel. Std. Dev.** | **Avg. Recovery Rate ± Std. Dev** |
| --- | --- | --- | --- | --- | --- | --- | --- | --- | --- |
|  |  |  | **1^st^ triplicate** | **2^nd^ triplicate** | **3^rd^ triplicate** |  |  |  |  |
| Mixed bread | Eurofins - IS | Ergocornine | 2.4 | 2.4 | 2.5 | **2.4** | 0.1 | 2% | 74 % ± 9 % |
|  |  | Ergocorninine | 1.7 | 1.8 | 1.7 | **1.7** | 0.0 | 1% | 59 % ± 2 % |
|  |  | Ergocristine | 4.3 | 4.6 | 5.2 | **4.7** | 0.4 | 9% | 46 % ± 19 % |
|  |  | Ergocristinine | 2.9 | 2.4 | 2.3 | **2.5** | 0.3 | 12% | 88 % ± 6 % |
|  |  | α/β-Ergocryptine | 3.9 | 3.9 | 3.6 | **3.8** | 0.2 | 4% | 62 % ± 8 % |
|  |  | α/β-Ergocryptinine | 3.3 | 2.9 | 3.3 | **3.2** | 0.2 | 7% | 77 % ± 5 % |
|  |  | Ergometrine | 0.9 | 0.8 | 1.0 | **0.9** | 0.1 | 10% | 99 % ± 6 % |
|  |  | Ergometrinine | 1.1 | 1.3 | 1.1 | **1.1** | 0.1 | 9% | 122 % ± 2 % |
|  |  | Ergosine | 3.0 | 3.0 | 3.5 | **3.1** | 0.3 | 11% | 74 % ± 7 % |
|  |  | Ergosinine | 2.2 | 2.0 | 2.6 | **2.3** | 0.3 | 15% | 52 % ± 27 % |
|  |  | Ergotamine | 6.7 | 5.4 | 5.4 | **5.8** | 0.8 | 13% | 84 % ± 16 % |
|  |  | Ergotaminine | 4.4 | 3.3 | 3.8 | **3.8** | 0.6 | 15% | 50 % ± 8 % |

**Table S29** Results for the quantification of the twelve priority ergot alkaloids in mixed bread using the standard addition method

| **Sample** | **Laboratory - Method** | **Analyte** | **Mass fraction [µg/kg]** | | | **Avg. Mass fraction [µg/kg]** | **Std. Dev. [µg/kg]** | **Rel. Std. Dev.** | **Avg. Recovery Rate ± Std. Dev** |
| --- | --- | --- | --- | --- | --- | --- | --- | --- | --- |
|  |  |  | **1^st^ triplicate** | **2^nd^ triplicate** | **3^rd^ triplicate** |  |  |  |  |
| Mixed bread | Eurofins - SA | Ergocornine | 2.5 | 3.0 | 2.9 | **2.8** | 0.3 | 10% | 63 % ± 2 % |
|  |  | Ergocorninine | 2.7 | 2.4 | 3.2 | **2.8** | 0.4 | 15% | 37 % ± 5 % |
|  |  | Ergocristine | 12.9 | 4.4 | 4.8 | **7.4** | 4.8 | 65% | 33 % ± 9 % |
|  |  | Ergocristinine | 2.9 | 2.4 | 2.3 | **2.5** | 0.3 | 12% | 64 % ± 4 % |
|  |  | α/β-Ergocryptine | 5.6 | 3.9 | 3.4 | **4.3** | 1.1 | 27% | 55 % ± 6 % |
|  |  | α/β-Ergocryptinine | 3.9 | 4.1 | 4.3 | **4.1** | 0.2 | 4% | 60 % ± 5 % |
|  |  | Ergometrine | 1.1 | 1.0 | 1.1 | **1.1** | 0.1 | 6% | 85 % ± 6 % |
|  |  | Ergometrinine | 1.2 | 1.5 | 1.3 | **1.3** | 0.1 | 10% | 105 % ± 1 % |
|  |  | Ergosine | 3.4 | 4.5 | 5.0 | **4.3** | 0.8 | 19% | 54 % ± 3 % |
|  |  | Ergosinine | 12.5 | 2.8 | 4.0 | **6.4** | 5.3 | 83% | 21 % ± 6 % |
|  |  | Ergotamine | 8.6 | 9.6 | 9.4 | **9.2** | 0.6 | 6% | 52 % ± 3 % |
|  |  | Ergotaminine | 15.8 | 5.3 | 6.6 | **9.2** | 5.7 | 62% | 24 % ± 7 % |
